# Care Under Pressure 2: a realist synthesis of causes and interventions to mitigate psychological ill health in nurses, midwives and paramedics

**DOI:** 10.1136/bmjqs-2023-016468

**Published:** 2024-04-04

**Authors:** Cath Taylor, Jill Maben, Justin Jagosh, Daniele Carrieri, Simon Briscoe, Naomi Klepacz, Karen Mattick

**Affiliations:** 1 School of Health Sciences, University of Surrey, Guildford, UK; 2 Exeter Medical School, University of Exeter, Exeter, UK; 3 School of Health Sciences, University of Southampton, Southampton, UK

**Keywords:** Health services research, Health policy, Leadership, Mental health, Organizational theory

## Abstract

**Background:**

Nurses, midwives and paramedics comprise over half of the clinical workforce in the UK National Health Service and have some of the highest prevalence of psychological ill health. This study explored why psychological ill health is a growing problem and how we might change this.

**Methods:**

A realist synthesis involved iterative searches within MEDLINE, CINAHL and HMIC, and supplementary handsearching and expert solicitation. We used reverse chronological quota screening and appraisal journalling to analyse each source and refine our initial programme theory. A stakeholder group comprising nurses, midwives, paramedics, patient and public representatives, educators, managers and policy makers contributed throughout.

**Results:**

Following initial theory development from 8 key reports, 159 sources were included. We identified 26 context–mechanism–outcome configurations, with 16 explaining the causes of psychological ill health and 10 explaining why interventions have not worked to mitigate psychological ill health. These were synthesised to five key findings: (1) it is difficult to promote staff psychological wellness where there is a blame culture; (2) the needs of the system often over-ride staff psychological well-being at work; (3) there are unintended personal costs of upholding and implementing values at work; (4) interventions are fragmented, individual-focused and insufficiently recognise cumulative chronic stressors; and (5) it is challenging to design, identify and implement interventions.

**Conclusions:**

Our final programme theory argues the need for healthcare organisations to rebalance the working environment to enable healthcare professionals to recover and thrive. This requires high standards for patient care to be balanced with high standards for staff psychological well-being; professional accountability to be balanced with having a listening, learning culture; reactive responsive interventions to be balanced by having proactive preventative interventions; and the individual focus balanced by an organisational focus.

**PROSPERO registration number:**

CRD42020172420.

**WHAT IS ALREADY KNOWN ON THIS TOPIC**

- Psychological ill health is prevalent in healthcare staff, particularly nurses, midwives and paramedics, and despite a plethora of interventions the problem persists.
- Previous reviews have focused on individual professions and/or individual-focused interventions.

**WHAT THIS STUDY ADDS**

- By contrast, the realist lens used in this study has illuminated tensions between aspects of healthcare work delivery which may be incompatible with maintaining healthy psychological states in healthcare staff, for example balancing and prioritising staff needs with service and patient needs, highlighting that healthcare delivery is a complex and dynamic balancing act.

**HOW THIS STUDY MIGHT AFFECT RESEARCH, PRACTICE OR POLICY**

- Psychological ill health in the healthcare workforce can be chronic and cumulative as well as acute and should be anticipated and prepared for.
- There is an urgent need to rebalance and refocus work efforts (in research, practice and policy) on multilevel systems approaches that take account of the often-conflicting interests between serving patients and protecting staff well-being.
- Developing context-sensitive approaches can help customise interventions given diversity within the workforce and structural differences between and within professions.

## Introduction

Health service delivery requires healthy motivated staff to provide high-quality patient care.^1–3^ The COVID-19 pandemic has shone a spotlight on the extreme challenges of healthcare work and the psychological ill health that can ensue. Yet, while the pandemic provided an intense and risky working environment, psychological ill health has been a considerable problem worldwide for many decades, leading to presenteeism, absenteeism and loss of healthcare staff from the workforce.^4–6^

The UK National Health Service (NHS) is the biggest employer in Europe and the world’s largest employer of highly skilled professionals, with 1.6 million employees.^4^ Nurses, midwives and paramedics comprise approximately 30% of the total workforce and over half of the clinical workforce.^5^ NHS staff are more likely to incur a work-related illness or injury than staff in other sectors,^7 8^ with higher rates of sickness absence compared with the average UK worker.^9^ Stress among healthcare staff is greater than in the general working population and explains more than 25% of absences. In the 2022 NHS Staff Survey^10^ 45% of staff reported feeling unwell due to work-related stress in the last 12 months, 57% of staff reported going to work despite not feeling well enough to perform their duties and 34% of staff stated they felt burnt out because of their work. The rates are among the highest in nurses, midwives and paramedics.

Multiple reports have highlighted the need to reduce stress and improve psychological health in NHS staff, recognising high financial and personal costs.^4 11–13^ Poor psychological ill health is estimated to cost the NHS £12.1 billion a year.^14^ Evidence suggests staff well-being at work is associated with both patient experience and safety outcomes.^15^ Work investigating this further suggests three workplace conditions—staffing for quality; psychological safety, teamwork and speaking up; and staff health and well-being at work—are essential to improving quality and safety in healthcare.^15^

There is a large body of literature on interventions that offer prevention, support or treatment to nurses, midwives and/or paramedics experiencing psychological ill health,^16–19^ yet the problem of psychological ill health of the workforce remains. Much evidence is profession-specific and/or does not take account of the complex context of the healthcare work environment, which limits understanding of the causes of (and solutions to) psychological ill health.

Therefore, our review aimed to answer the following questions: how, why and in what contexts (a) do nurses, midwives and paramedics experience work-related psychological ill health and (b) are existing interventions insufficient to mitigate it.

## Methods

### Design

We undertook a realist synthesis adhering to the RAMESES (*R*ealist *A*nd *Me*ta-narrative *E*vidence *S*yntheses: *E*volving *S*tandards) guidelines^20^ (online supplemental appendix 1). The protocol is published in full on the funder’s website (https://fundingawards.nihr.ac.uk/award/NIHR129528) and registered with PROSPERO (ID: CRD42020172420). Realist synthesis is a theory-driven approach that seeks to answer ‘what works, for whom, how and in what circumstances/context’. Previous reviews of psychological ill health in healthcare workers have tended to focus either on causes (eg, refs 21 22) or interventions (eg, refs 23–25) (rather than both combined) and have not prioritised consideration of contextual factors impacting on how psychological ill health develops or how an intervention works or not.^16^ A realist synthesis places context centre-stage to take account of organisational and structural contexts (eg, specialty, setting, culture and policies, economic and wider societal factors^26^) and explore profession-specific working practices (eg, shift work, team or lone working) and similarities and differences in organisational factors, context and working practices (which we call ‘service architecture’). Exploring tensions between different aspects of work for healthcare employees, we were able to develop a programme theory to explain how these might influence the development of psychological ill health and the uptake and success of interventions aimed at supporting psychological wellness. See box 1 for a glossary of terms.

10.1136/bmjqs-2023-016468.supp1 Supplementary data

Box 1 **Glossary of terms**

- **Appraisal journalling**: creation of journal entries for each paper that addresses (a) the important insights described or inspired from the document in relation to the overall analysis and (b) team member journal-on-journalling to build coproductive analysis.
- **COVID-19**: a highly contagious respiratory disease caused by the SARS-CoV-2 virus. The disease SARS-CoV-2 causes is called COVID-19.
- **Context–mechanism–outcome configurations**: relationships between the building blocks of realist analysis (ie, how mechanisms are triggered under specific contexts to cause particular outcomes).
- **Contexts**: settings, structures, environments, conditions or circumstances that trigger behavioural and emotional responses (ie, mechanisms) in those affected.
- **Mechanisms**: the way in which individuals and groups respond to and reason about the resources, opportunities or challenges offered by a particular programme, intervention or process. Mechanisms are triggered in specific contexts and lead to changes in behaviour, and consist of the resource offered and the reasoning response to the resource.^170^
- **Outcomes**: impacts or behaviours resulting from the interaction between mechanisms and contexts.
- **Programme theory** (initial and final): a set of theoretical explanations or assumptions about how a particular programme, process or intervention is expected to work.
- **RAMESES guidelines**: *R*ealist *A*nd *Me*ta-narrative *E*vidence *S*yntheses: *E*volving *S*tandards guidelines to ensure rigour.
- **Realist synthesis**: the analysis of a wide range of evidence that seeks to identify underlying causal mechanisms and explore how they work under what conditions, answering the question ‘what works for whom under what circumstances?’ rather than ‘what works?’
- **Retroduction**: identification of hidden causal forces that lie behind identified patterns or changes in those patterns; or retroductive: the activity of uncovering underpinning mechanisms.
- **Reverse chronology quota sampling**: working backwards in date order from the most recent relevant publications until a predetermined set number (quota) of papers had been met.
- **Service architecture**: the way work is organised—the organisational factors, context and working practices.
- **Tensions**: aspects of work that are incompatible with each other and affect psychological ill health.

### Stakeholder/patient and public involvement and engagement group

A stakeholder group comprising nurses, midwives, paramedics (including lived experience of psychological ill health at work) and members of the public supported the testing and refinement of the final programme theory through four meetings during the project. Stakeholder meetings enabled us to test the resonance of our emerging theory and to gain wider input to refine and extend our developing programme theory. The stakeholder group also helped refine the terminology of psychological ill health applied in this study.

#### Terminology

Our review built on Care Under Pressure 1 (CUP1)^27^ and used similar inclusion and exclusion criteria and conceptualisation. However, in a stakeholder meeting, the term mental ill health was felt to be stigmatising and alienating, aligning with clinical diagnoses rather than the broader conceptualisation we were seeking. Therefore, we use psychological ill health to avoid pathologising mental ill health and encompass common psychological ill health problems (eg, stress, distress, anxiety, depression) and both proximal (eg, retention, absenteeism, resilience) and distal endpoints (eg, burnout), and also precursors to psychological ill health (eg, conflict, moral distress).

### Initial programme theory

Our starting point in developing our initial programme theory (IPT) was the final programme theory from CUP1.^28^ This comprised four intertwined clusters explaining the causes and interventions to mitigate mental ill health in doctors. Isolation was identified as a key cause, and beneficial interventions included those that considered group belonging and relationality, balanced prevention of ill health with promotion of psychological well-being, and were timely and implemented in a way that engendered trust. Our previous mapping of demographic, service architecture and psychological well-being indicators across nurses, midwives, paramedics and doctors^5^ indicated key differences that may relate to the causes and consequences of work for psychological health, including gender and age profile differences, higher salary in doctors, and higher sickness absence and presenteeism in nurses, midwives and paramedics. We also undertook preliminary reading of key literature.^8 16 17 29–32^ Our initial assumptions about how, why and in which contexts nurses, midwives and paramedics experience work-related psychological ill health, despite interventions to mitigate it, were the following:

1. Psychological ill health is prevalent across all healthcare professions, and differences between the professions and the way they work suggest there are profession-specific causes.
2. Thus, interventions may need to be profession-specific to be effective.
3. The CUP1 final programme theory is likely to be relevant to nurses, midwives and paramedics, but other aspects of work and explanations may be important due to professional structural and demographic differences.

Interventions in the literature focus on individuals and are therefore unlikely to address the organisational-level causes of psychological ill health. These elements of the IPT were considered and tested during the analysis of new evidence (see below).

### Searching and screening

Evidence was searched and screened for inclusion in the following iterative cycles:

1. Formal bibliographic database searching: this included (a) a search focused on each of the three professions and (b) an expanded paramedic search due to limited studies in the initial search. Three databases were searched in February 2021: MEDLINE All (via OVID), CINAHL (via EBSCO) and HMIC (via OVID). Search terms included terms for the population of interest (nurse, midwives and paramedics), common psychological ill health problems (eg, stress, anxiety, depression) and outcomes of psychological ill health (eg, sick leave and burnout). To maintain relevance to the UK NHS’ context, we limited searches to UK-based literature using a published search filter for MEDLINE^33^ and a search function within CINAHL. An additional search with more sensitive search terms informed by paramedic stakeholders (including ‘first responder’ and ‘emergency personnel’) and a published search filter for the paramedic field^34^ was run on 31 March 2021. The MEDLINE search is included in online supplemental appendix 2. For screening, we used reverse chronology quota (RCQ) sampling to work backwards from the most recent relevant publications for each profession until a predetermined quota of papers was met. This ensured roughly equal numbers of papers for the initial round (~30 per profession), ensured the review was manageable in scale and enabled a focus on the most up-to-date evidence, theories and frameworks. We excluded the following papers: (a) physical health focus, (b) undergraduate students, (c) non-UK, (d) patient well-being and (e) published prior to 2010 or prior to the 30 most recent relevant papers .
2. Supplementary handsearching: we searched back issues of the *British Midwifery Journal*, *Journal of Paramedic Practice* and *British Paramedic Journal*, starting from the most recent edition and applying the same exclusion criteria.
3. Expert solicitation: to address the potential limitation of RCQ missing major insights in earlier published literature, we sought relevant papers through expert solicitation with team, stakeholder and advisory group members.
4. COVID-19-specific literature: this search was run in 2021 and used the same professional and mental ill health terminology as the initial search in step 1, but replaced search terms for the outcomes of mental ill health with COVID-19 search terms developed by the UK Health Security Agency Library Services team.^35^ Using a COVID-19 filter enabled a more sensitive search for COVID-19 literature which was not limited to the outcomes of mental health as per step 1. We secured a number of COVID-19 papers via expert solicitation to add ‘in press’ and more recent literature given the timing of this review. For all searches, two team members (JJ and CT) independently screened papers for inclusion based on assessment of relevance and rigour, with disagreements arbitrated by a third team member (JM).

10.1136/bmjqs-2023-016468.supp2 Supplementary data

### Realist synthesis

We used appraisal journalling to analyse each source. This comprised reading and annotation by the lead reviewer (JJ), who then ‘journalled’ important insights in a working document alongside the abstract. The wider team then read the paper (at minimum the abstract) and the lead reviewer’s insights/thoughts and added further insights, drawing on their disciplinary and NHS expertise, providing challenge and counterarguments and reflecting on ‘fit’ to the initial programme as well as new theory and ideas. Initial syntheses of the journalling were based on a small sample of papers (n=15, 5 for each profession), with subsequent papers journalled in batches and folded into the analysis.

These cumulative and collaborative insights were used to formulate context–mechanism–outcome configurations (CMOcs) to describe how/why/for whom and in what circumstances (a) nurses, midwives and paramedics experience work-related psychological ill health and (b) existing interventions are insufficient to mitigate it. At an early stage, we began to identify aspects of work that were antagonistic and incompatible and affected staff psychological health. We called these ‘tensions’ in the work environment and they provided a framework for our analyses, enabling us to synthesise CMOcs to a higher level and to go beyond a superficial view of the evidence. We shared emergent analyses with the stakeholder group to sense-check the emerging findings, help us determine the novel and most important findings, and generate further insights into the impact of these tensions on psychological ill health and on the effectiveness of interventions designed to mitigate against it. The final programme theory synthesised the data to five higher-level statements, underpinned by the tensions and CMOcs.

Diversions from the protocol were minimal but included the following: (a) only including MEDLINE, CINAHL and HMIC in the initial database search (not PsycINFO, Cochrane Library or ASSIA), and not systematically undertaking forward and backward citation—this was due to the rich insights found through the additional methods of handsearching and expert solicitation, as recommended by stakeholders who stated there were few select journals that would be valuable to search; and (b) neither RCQ screening nor appraisal journalling was mentioned in the protocol, but were strategies employed to manage the high volume of papers and support the integration of ontologically deep analysis of papers with team member expertise.

## Findings

### Realist synthesis: key findings

**Figure 1:**
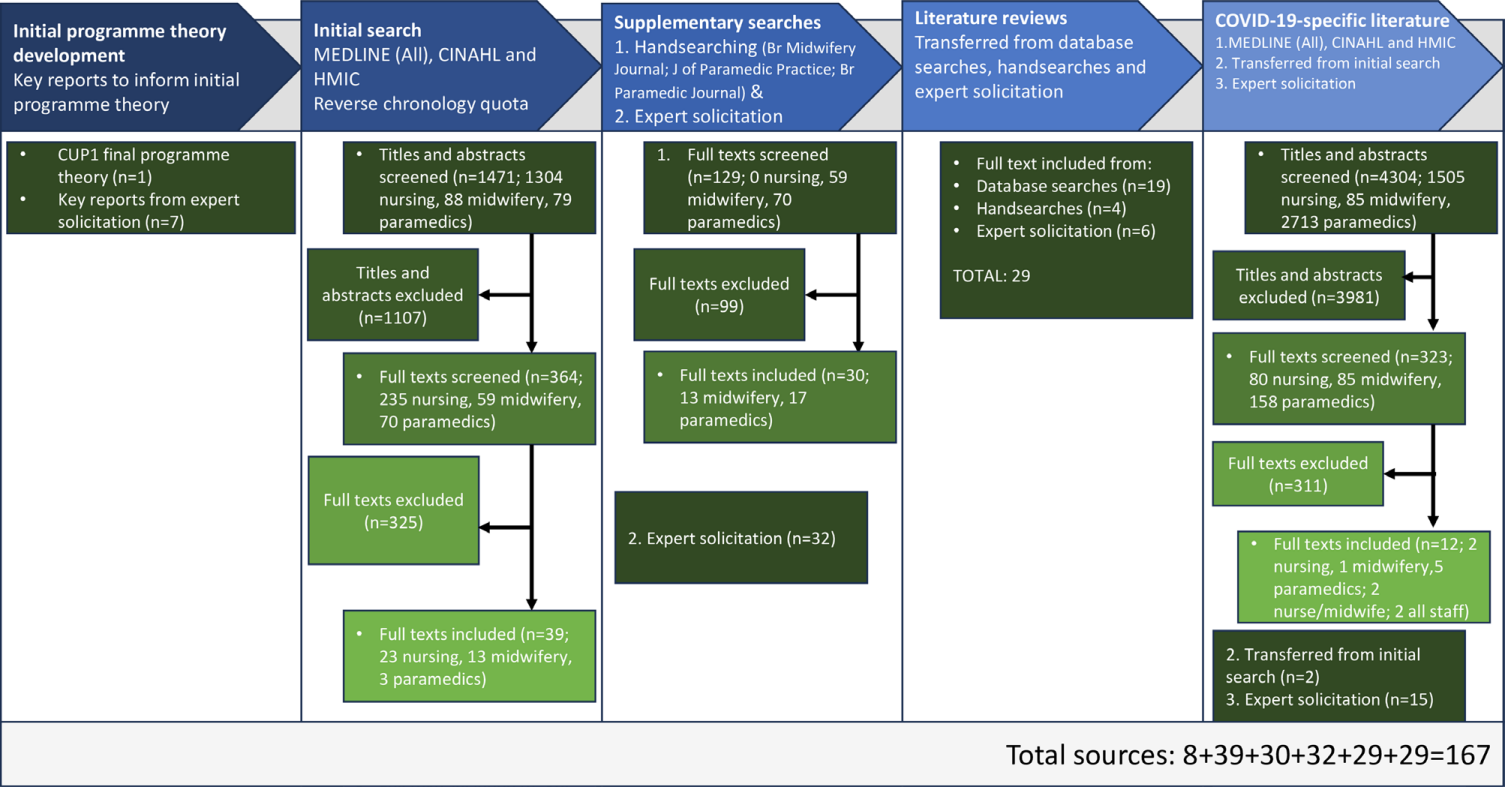
RAMESES flow diagram showing the sequence of steps in the review. CUP1, Care Under Pressure 1; RAMESES, *R*ealist *A*nd *Me*ta-narrative *E*vidence *S*yntheses: *E*volving *S*tandards guidelines.

Following IPT development and review of 8 key reports, 159 papers were included: 39 in the first round, 62 in the second (30 from handsearching, 32 expert solicitation papers), 29 literature reviews and 29 COVID-19-specific papers. Thus, after iterative cycles of searching and synthesis, a total of 167 sources were included (online supplemental appendix 3). See the RAMESES flow chart of the search and screening process (figure 1). Sources included empirical studies (quantitative, n=20; qualitative, n=34; mixed methods, n=7), commentaries (n=30), editorials (n=18), discussion papers (n=7), grey literature mixed methods study (n=1), continuing professional development papers (n=2) and review articles (n=40).

10.1136/bmjqs-2023-016468.supp3 Supplementary data

Our realist analyses identified 26 CMOcs that explained 14 key tensions. These were synthesised to five key findings (table 1, online supplemental appendix 4), described in the following section, which led to our final programme theory.

10.1136/bmjqs-2023-016468.supp4 Supplementary data

**Table 1:** Key findings, tensions and exemplar CMOcs

Key finding	Tension	CMO	Context–mechanism–outcome configuration
It is difficult to promote staff psychological wellness where there is a blame culture.	A lack of collective accountability vs a team/system-based approach.	1	Front-line staff are most directly linkable to outcomes and errors (C); a focus on performance measurement and accountability of individual staff can preclude acceptance of system-wide accountability (M-resource), leading to staff practising defensively to avoid blame (M-response), reducing job satisfaction and increasing risk of secondary trauma (O).
	Needing to raise concerns vs fitness-to-practise processes felt as being oppressive.	5	When medical errors occur in an organisation where staff do not feel psychologically safe (C), investigation of errors (M-resource) may make staff feel unheard or blamed, and they may fear public exposure and reputational damage and not able to speak up, and instead feel guilt and shame (M-response), leading to silencing, frustration and secondary trauma (O).
	Encouraging staff to speak up vs the ‘deaf effect’ response from managers and hearers.	6	When it is not psychologically safe to speak up about mistakes or where senior leaders do not listen to staff concerns (C), when encouraged to speak up and raise concerns (M-resource), staff will fear the consequences or feel there is no point as no change will result (M-response), leading to decreased workplace satisfaction, reduced quality of patient care and increased secondary trauma (O).
‘Serve and sacrifice’: the needs of the system often over-ride staff well-being at work.	A culture in which staff prioritise institutional needs vs a culture that promotes self-care.	8	When high workloads are normalised in professions that are exhorted to put patients first (C) and if staff are told to give 100% to serve patients without providing support strategies (M-negative resource), this reinforces compliance to institutional needs to the detriment of staff needs (M-response), leading to guilt and increased stress, burnout and intention to leave/attrition (O).
	Supporting staff in the context of staff shortages vs the need to fill ‘extra’ vacant shifts.	9	Managers feel pressure to ensure safe staffing levels despite staff shortages (C); if managers communicate this pressure to staff ‘begging’ them to work extra shifts (M-negative resource), staff can feel coerced and/or guilty when they say no (M-response), preventing non-work time from being regenerative, leading to increased job dissatisfaction, presenteeism and burnout (O).
	The lived reality of staff shortages vs the wish to deliver high-quality care.	10	Staff shortages mean there is less time to care for each patient (C) and staff cannot provide their preferred quality of care (M-negative resource), leaving them feeling frustrated, angry and guilty at care left undone (M-response), leading to moral distress, burnout and intention to leave/attrition (O).
There are unintended personal costs of upholding and implementing values at work.	The reality of healthcare delivery vs the taught theory and values.	13	If newly qualified staff have developed idealised visions of work (C), then when pressures caused by systemic factors mean their practice may not align with such ideals (M-negative resource), they may feel moral distress (M-response), causing reduced job satisfaction, burnout and attrition (O).
	The benefits of staff empathy to patients vs the harms of such empathy to themselves.	14	Staff are recruited based on values, including compassion (C); when they are genuinely empathic (M-resource), they are better able to understand patients’ pain/suffering (M-response1), leading to better patient care and increased job satisfaction (O1), but empathising with patient suffering may cause staff distress (M-response2), leading to burnout, secondary trauma and attrition (O2).
	The emotional labour required for health work vs protecting staff’s psychological ill health.	16	Healthcare staff may be exposed to injuries or suffering that evokes natural emotions such as repulsion, fear or distress (C), but have to repress responses to protect patients (M-resource), which can lead to emotional distress in staff (M-response), causing suppressed emotions to come out in other dysfunctional ways, impacting job satisfaction, performance and psychological health (O).
Interventions are fragmented, individual-focused and insufficiently recognise cumulative chronic stress.	A focus on individuals vs a focus on systemic issues.	18	When there is normalisation of unpaid overtime and an absence of a systemic focus on well-being (C), if leaders encourage staff to prioritise self-care (M-resource) staff may feel their leaders are out of touch with reality (M-response), leading to reduced job satisfaction, work engagement and morale (O).
	A focus on acute trauma episodes vs recognising and supporting chronic cumulative stressors.	20	Constant low-grade trauma exposure to patient suffering, resource scarcity and staff shortages may not be visible (C), meaning that managers may not recognise the cumulative build-up of stress (M-negative resource) and may thereby judge staff competency unfairly (M-response), causing increased stress, risk of secondary trauma and intention to leave/attrition in staff (O).
It is challenging to design, identify and implement interventions.	Making staff wellness interventions mandatory vs making them voluntary.	22	When prioritising staff well-being (C), attendance at a wellness intervention may be mandated when some staff are not receptive to it (M-resource), leading to staff feeling that the approach is a tick box and lacking authenticity, and then feel resentful, anxious, exposed or stigmatised at sharing emotions (M-response), causing staff work disengagement and feeling less secure/likely to speak up (O).
	Spaces to debrief with leaders vs peer-led spaces for debriefing.	25	Healthcare staff may be exposed to chronic and acute trauma (C); if mentors offer kindness and spaces to be heard (M-resource), staff feel their experiences are important and recognised (M-response), and are helped to recover, continue with work and protected from further harm (O).
	The need to offer support vs providing interventions too soon, too reactive and/or at a single timepoint.	26	If staff’s basic physiological and safety needs are not met (C), then when they are offered other support/interventions such as end-of-shift debriefing (M-resource) they may feel frustrated and upset due to the lack of recognition of their other essential needs, and fatigue and exhaustion due to intense working shifts preventing attendance (M-response), causing low uptake /engagement and exacerbation of distress/trauma response (O).

see online supplemental appendix 4 for all CMOcs.

CMOcs, context–mechanism–outcome configurations.

#### It is difficult to promote staff psychological wellness where there is a blame culture

A blame culture is ‘a set of norms and attitudes within an organization characterized by an unwillingness to take risks or accept responsibility for mistakes because of a fear of criticism or management admonishment’ (p314–315).^36^ It is the opposite of a psychologically safe culture and prevents people from speaking up and taking accountability.^15^ Healthcare is a complex workplace and human error is inevitable, yet front-line staff can sometimes be linked directly to service outcomes and medical errors, precluding an acceptance of system-wide accountability. This can lead staff to practise defensively to protect themselves from blame (eg, refs 29 37 38). Furthermore, mistakes can lead to staff being ‘second victims’, feeling shame, guilt, panic, shock and humiliation, leading to self-doubt and loss of confidence, and potentially to further errors and/or leaving the profession^29 37 39 40^ (CMO1). Healthcare work can feel interpersonally risky if it is not psychologically safe to ask questions or admit a mistake (and risk looking incompetent)^15 41 42^ (CMO5). Alternatively, when they feel safe to be open and honest, staff are willing to speak up and learn from mistakes, leading them to feel supported and improving learning and performance in individuals and organisations.^43^

We found evidence that being investigated for medical errors can cause psychological ill health in staff (eg, refs 44 45), with calls for compassion and fairness in such processes.^8^ The lack of attention by regulatory bodies and NHS organisations to wider context (eg, understaffing, toxic work environments) can mean investigations focus on the individual rather than the wider system. This can lead staff to feel guilty, unsupported and isolated, and can lead to increased secondary trauma and potentially suicidal ideation, with resulting trauma extending to family and friends^8 17 45–48^ (CMO3). Furthermore, perceptions that fitness-to-practise processes are unsupportive create reluctance in staff to voice concerns about their psychological health, which can therefore remain undisclosed, increasing risks to staff and patients. This represents a missed opportunity to create a culture of shared learning, transparency and reflection and to de-stigmatise psychological health issues^8 45 49 50^ (CMO4).

The literature contained evidence of double standards in accountability: for example, staff having to work in clinical areas known for poor standards of care and being held individually accountable, yet managers not being held accountable to fix known issues^51^ (CMO2). This may be due to the organisation perceiving itself as a ‘third victim’ due to potential negative financial and reputational impacts of errors,^37^ or to conflict between managers’ roles in performance management and emotional support,^37 52^ affecting the ability to listen to staff and learn from mistakes. Initiatives such as ‘Freedom to Speak Up Guardians’, designed to encourage staff to speak up or raise concerns that require action, may be ineffective in contexts that are not psychologically safe, especially if senior leaders do not listen (described as the ‘deaf effect’)^41 53–55^ (CMO5 and CMO6). This may also apply to other discursive/speaking-up interventions, such as various models of clinical supervision including resiliency-based supervision.^56 57^ Unless the organisational and structural causes of psychological ill health at work are recognised, such interventions may ‘backfire’, incurring unintentional harms to staff and increasing mistrust in the organisation (eg, ref 57) (CMO7).

#### ‘Serve and sacrifice’: the needs of the system often over-ride staff psychological well-being at work

Nurses, midwives and paramedics are exhorted to put patients first, often putting their own needs second. This can erode psychological well-being in the face of intense and potentially traumatic work, and (counterintuitively) compromise high-quality patient care.^40 58 59^ High workload can become normalised and rest breaks sacrificed.^8^ The message that healthcare professionals should give 100% to patients, in a context where there are few strategies to help staff manage complex/distressing clinical situations, may reinforce compliance to institutional needs but to the detriment of staff’s psychological health^58 60^ (CMO8). This was amplified during the pandemic^61–65^ with normalisation of a ‘serve and sacrifice’ approach.^63 66 67^

A further tension emerged between promoting staff psychological well-being in the context of staff shortages. This includes the pressure felt by staff when managers ask (or sometimes beg) them to take on extra shifts and staff feeling coerced to say ‘yes’, leading to feelings of guilt when they are not working^68^ (thus time off work is not regenerative) (CMO9). This is particularly relevant to nurses, midwives and paramedics working 12-hour shifts, since long shifts mean they have several days off that may be seen as ‘available for work’.^69^ This can be exacerbated in individuals who have few interests and activities outside work,^70^ and in organisations with cultures that exalt loyalty, teamwork and professional identity, which can ‘unwittingly encourage presenteeism’ (p2).^71^

Staff shortages also have a direct impact on patient care quality. Where staff cannot provide the care quality they feel patients deserve, this can result in moral distress and injury; feelings of anger, frustration and guilt; burnout and dissatisfaction with work; and decisions to leave the profession^8 72–75^ (CMO10). A vicious cycle may arise whereby under-resourced work environments lead to unworkable situations for those who remain, who may ultimately also choose to leave^31 76^ (CMO11). Staff shortages can also lead to overextensions of the role scope for those who remain, leading to anxiety about quality, thereby increasing the risk of psychological ill health, sickness absence and intention to leave the profession. This was amplified during COVID-19 with staff being redeployed^29 71 77 78^ (CMO12).

#### There are unintended personal costs of upholding and implementing values at work

Healthcare staff are educated to hold strong professional values and codes of conduct, including being compassionate and empathic. Evidence suggests that a caring healthcare interaction is highly associated with patient satisfaction,^79^ better patient outcomes^80 81^ and healing.^82 83^ However, providing compassion and empathy to patients in the context of physical and emotional exhaustion can impact negatively on staff psychological health, resulting in vicarious or secondary trauma^8 84 85^ (CMO14). Literature suggests that some of the traits that attract individuals to healthcare work (eg, the ‘desire to help’) may contribute to vicarious trauma.^86^ Being frequently exposed to traumatic events, lacking time to process experiences and working in an unsupportive workplace environment increase the risk of harm from providing empathic compassionate care.^87^

Moreover, to deliver compassionate high-quality care, emotional labour is required; yet nurses, midwives and paramedics often have to suppress authentic feelings and regulate their emotions, which may include ‘turning down the volume’ on empathy (ie, keeping an emotional distance from patients^88^) or taking a problem-solving approach devoid of empathy^89^ to cope. This may result in the rewards of providing care being missed, leading to decreased work satisfaction and reduced personal accomplishment (an element of burnout).^89–91^ Previous literature suggests such strategies are defences against anxieties caused by work.^92^ Healthcare staff may also have to regulate emotions to provide hope and positivity to patients and family, and temporarily hide emotions such as revulsion, fear or distress.^93^ This may evoke feelings of trust, reassurance and hope in patients, but may lead to maladaptive outlets for staff, including use of dark humour,^94–97^ but also alcohol, drugs, etc, if there are no formal or informal outlets at work^40 98–101^ (CMO15 and CMO16).

A further tension identified was the theory–practice gap between theory taught in healthcare education and the reality of healthcare delivery.^74 102 103^ If newly qualified staff cannot deliver care in line with their idealised vision of a ‘good’ healthcare practitioner, this can cause guilt and moral distress or injury, causing them to leave^104–106^ (CMO13). More experienced staff may have had to either compromise their ideals of care, job-hop or leave the profession due to their ideals being compromised or crushed.^72 88^ During the pandemic, the ‘deathscapes’ and tragic choices that staff had to make exemplified this.^62 98 107–111^

#### Interventions are fragmented, individual-focused and insufficiently recognise cumulative chronic stressors

Identified interventions were fragmented rather than synergistic and systemic, and many were individual-focused, aiming to fix or reduce risk in individuals by modifying their behaviour or responses to stressors, rather than change the workplace environment and/or be preventative.^29 112 113^ This focus suggests protecting psychological well-being is solely the responsibility of individuals (with little or no organisational responsibility), which may result in staff feeling blamed for feeling stressed/distressed, rather than acknowledging that systemic issues may be the root cause. 18 114–120 Offering individual-focused interventions in the absence of addressing wider systemic issues can be perceived as blaming staff for not being resilient enough in the face of insufficient support and/or resources (eg, refs 16 32) (CMO17). Furthermore, when staff are exhorted to put patients first and hide their needs and emotions, it is challenging for them to undertake self-care.^121^ The evidence highlighted the importance of leaders/managers giving staff permission to be self-compassionate (eg, refs 29 122), yet such messaging may backfire if at odds with the reality of work conditions, leading the staff to feel managers are failing to acknowledge the serious negative impacts of under-resourced work environments, resulting in decreased job satisfaction and reduced work engagement and morale (eg, refs 17 40) (CMO18). Role-modelling of self-care by individual leaders, and organisationally, was identified as important, for example, through implementation and prioritisation of interventions that put staff experiences first, such as Schwartz Rounds^123–125^ (CMO19).

Psychological ill health was often implicitly conceptualised in a binary form (ill or well), rather than acknowledging its fluctuating state, and focused on acute event impact rather than the cumulative impact of everyday healthcare work^126 127^ and the need for long-term support post-COVID-19.^66 128–130^ The impact of prolonged exposure to poor working conditions, such as low staffing, poor skill mix, unpaid overtime and steadily increasing work pressures,^22^ was less frequently discussed, with one paper recognising that ‘it may take a while for the impact of these demands to manifest in terms of symptoms’ (p575)^131^ (CMO20). Another described the behaviour and work practice microadjustments made to cope with pressures and the consequent normalisation of such behaviours.^132^ Relatively minor incidents may trigger big reactions: the ‘final straw’ in a long string of experiences involving secondary trauma^133^ (CMO21). Contextual features may also increase risk of secondary traumatic stress or distress, such as incidents involving children,^40 134^ or where staff particularly connect to the patient or incident.^16^

#### It is challenging to design, identify and implement interventions to work optimally for diverse staff groups with diverse and interacting stressors

Our research highlighted the challenges of designing and embedding complex interventions within large organisations that meet the dynamic needs of diverse groups of healthcare staff. Previous research with doctors^28^ and evidence-based implementation science frameworks (eg, refs 135 136) suggest endorsement, expertise, engagement and evaluation are important factors for successful implementation. Interventions are most likely to work when tailored to specific contexts and needs of the staff group(s), and when staff are engaged in intervention development (codesign), shaping and implementing changes.^137^ Our research identified the importance of considering who, when and how interventions are delivered, and not just what is being delivered, and a specific focus on intersectionality factors (eg, ethnicity, disability^138 139^) is required.^8^

Tensions we identified included whether staff psychological wellness interventions should be mandatory versus voluntary. Making interventions mandatory may enable them to be normalised and help change culture regarding support for the emotional impacts of work,^140 141^ but may lead some staff to feel resentful, anxious and exposed when sharing emotions. It may also retraumatise staff by requiring disclosure to others^72 142 143^ (CMO22). A further risk of mandating attendance is that it is perceived as a ‘tick box’ response, with the intervention seen as simply a management tool^72 144^ (CMO24). Offering debriefs or check-ins as a voluntary/optional intervention means those who wish to discuss/receive support can do so, but may result in others who need support not accessing it due to fear of stigma^72 112 142 145^ or not recognising the benefits (CMO23).

Another tension was the need to act and offer support versus providing interventions that are ineffective because they are too reactive and/or at a single timepoint. There was significant learning about the type and timing of interventions from the COVID-19 pandemic. For example, barriers to access the range of interventions on offer included time constraints, physical barriers (eg, geographical distance), no access to resources at work and no desire to access outside working hours.^146 147^ A key issue was that they were often not the right interventions at the right time; staff needed their essential safety and physiological needs met first (access to food, drink, breaks),^42 146 148 149^ followed by potential access to psychological support once the threat receded. Getting the timing wrong can lead to low uptake and thereby exacerbation of distress (CMO26).

The involvement, or not, of supervisors/managers in supporting the processing of work challenges was another tension. Formal debriefing following trauma exposure (eg, via occupational health departments, or using immediate or delayed debriefs^150 151^) can provide opportunity to process difficult experiences,^152^ but may not work if perceived as a management tool (CMO24). Managers having a relational leadership style,^153^ listening and offering kindness and spaces to be heard can reinforce that staff are valued and their experiences are valid and recognised, and help staff to recover and feel less alone^154^ (CMO25). On the contrary, peer-led spaces for debriefing in a confidential psychologically safe space can bring safety and willingness to disclose difficulties that might not be possible with managers.^107 155–159^ However, managers/leaders might then be unaware of issues and unable to act and signpost to support.^160^ There is a need for interventions aimed at both organisational learning and staff healing (eg, peer-led informal spaces and countercultural organisation-wide spaces such as Schwartz Rounds). The importance of spaces and places for staff to come together to meet, off-load and listen was clear. Such spaces have been eroded over time, with staff break rooms being non-existent or having multipurpose functions,^145^ and some service architecture features make informal peer support challenging (eg, lone workers).^161–163^

### Final programme theory

**Figure 2:**
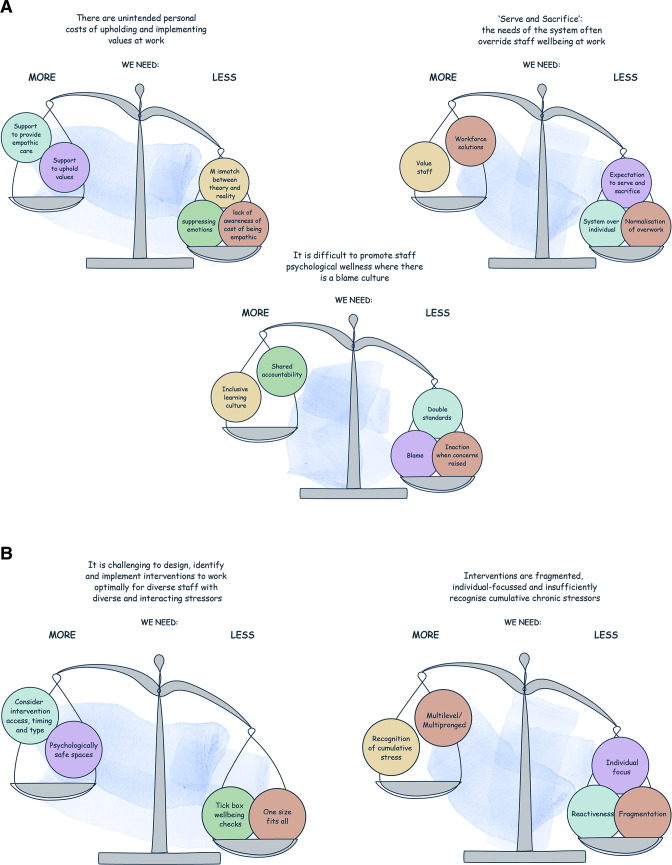
(A) Imbalances in the work environment explaining the causes of psychological ill health in nurses, midwives and paramedics. (B) Imbalances in the work environment explaining why interventions do not work.

**Figure 3:**
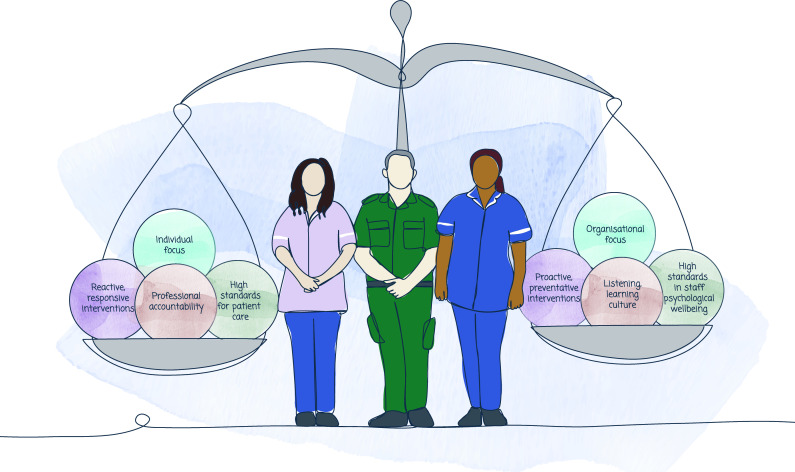
Final programme theory: key focus areas to restore the balance.

The tensions (aspects of work that appear incompatible and affect psychological ill health) identified through our analysis showed that healthcare provision is a balancing act, with different considerations needing to be held in productive tension, such as the needs of staff and the needs of patients (figure 2A,B). Our final programme theory identifies the key areas required to restore balance and support psychological well-being in healthcare staff (figure 3), where elements on the right require focus and attention to rebalance with those on the left.

## Discussion

While there have been many previous reviews examining the causes of, or interventions to mitigate, psychological ill health in healthcare staff, critically high levels of psychological ill health remain. Our realist approach adds significantly to the literature on the psychological ill health of nurses, midwives and paramedics, highlighting the importance of context and uniquely identifying key tensions (incompatible aspects of work) affecting psychological health, supported by causative explanations rooted in the literature.

We propose a focus on four key areas to restore the imbalances we report. First, healthcare organisations have a duty to protect and deliver high-quality care to patients, yet this needs to be rebalanced against healthcare organisations as employers, with a duty to protect employees and provide an environment where staff can thrive. Fundamental to staff psychological well-being—supporting Maslow’s hierarchy of needs theory^148^—is ensuring essential needs are met, such as having breaks and access to food and drink. In other safety critical industries, such as aviation and nuclear, ‘red rules’ denote safety rules that must not be broken due to the risk of harm. While ‘never events’ are used in healthcare, they are predominantly applied to patient safety^164^ or in relation to physical harm to employees (eg, infection) rather than psychological harm. This is despite provisions in the UK and international law that all workers are entitled to work in environments where risks to their health and safety are properly controlled. The risk of psychological harm to front-line healthcare workers is high and arguably inevitable, and as such should be anticipated and planned for. Hard hats and protective equipment are mandatory on building sites; something equivalent is required to protect the psychological well-being of healthcare workers and could be planned for on entry to the workforce rather than waiting for harm to occur. Risk assessment that considers the service architecture of an employee’s role (eg, working predominantly alone, likely exposure to acute trauma, etc) and higher-risk characteristics (eg, being newly qualified, going through complaints or investigations, intersectionality factors such as ethnicity, disability, etc) is key to this.

Second, while professional accountability is critical to ensure patient safety, this must be balanced with promoting listening, learning cultures.^8^ We found collective blame is often attributed to individual staff, with double standards in accountability and fitness-to-practise processes that can cause great harm, and when staff do speak up they encounter a ‘deaf effect’ with no action. A psychologically safe culture, where visible leaders enable and support staff and take accountability, is urgently required. Initiatives in the UK NHS, such as ‘Freedom to Speak up Guardians’, are promising but need adequate investment and boards willing to really listen to change culture.^165^

Third, the reactive and responsive interventions identified in the literature (predominantly focused on support following acute trauma) must be balanced with the development of proactive preventative interventions. Trauma can be chronic and cumulative, with seemingly benign (and thereby unnoticed) events triggering psychological ill health. Investment of time and funding for psychological well-being may reduce the stigma associated with experiencing burnout/stress by normalising it as an expectation of the job and enable anticipatory planning The appointment of well-being guardians in the UK NHS^8^ signals board-level leadership and responsibility for the psychological well-being of staff. This initiative, although welcome, requires adequate resourcing and evaluation.

Finally, an individual focus where staff may feel blamed for their own psychological ill health must be balanced with an organisational focus to address systemic issues. While individually focused interventions aimed at modifying response to stressors (such as mindfulness) may be useful in the moment, a multilayered systems approach to staff psychological well-being is needed, with organisation-wide interventions and bundles of support.^166–168^

We recognise the challenges and barriers to the development and implementation of our findings at a time of high demand, high sickness absence and staff shortages in the UK NHS and elsewhere. Yet, without renewed strategic focus and substantial intervention, the situation will surely worsen. We have developed a guide for policy makers, healthcare leaders, managers, and nurses, midwives, and paramedics providing practical tips and examples of how and where to intervene.^169^ We continue to work with national staff psychological well-being leads to bring about effective change, building on the evidence presented here.

### Strengths and limitations

Realist methodology enabled a depth of investigation not previously achieved, including uncovering previously unidentified tensions. The work was strengthened by the multidisciplinary expertise of the team, advisory and stakeholder groups (including staff experts by experience), which ensured relevance of the findings to real-world problems. The RCQ method we applied to manage the large literature (particularly nursing) may mean we missed key sources, although expert solicitation mitigated this risk. The searches were intentionally UK-focused, so the findings may not be transferable.

## Conclusion

Healthcare delivery is a balancing act, with fundamental tensions between being a care provider and an employer. Psychological ill health is highly prevalent in nurses, midwives and paramedics, developing through a number of complex and interrelated factors. Therefore, psychological ill health should be anticipated and prepared for, indeed normalised and expected. The working environment needs changing urgently to enable healthcare staff to recover and, ultimately, thrive.

## Data Availability

Data sharing not applicable as no data sets generated and/or analysed for this study.
