# Supplementary material for: Care Under Pressure 2: a realist synthesis of causes and interventions to mitigate psychological ill health in nurses, midwives and paramedics
Source: BMJ Qual Saf. 2024 Apr 4;33(8):523–38. doi: 10.1136/bmjqs-2023-016468 (PMC11287552; doi:10.1136/bmjqs-2023-016468)
Supplement: Supplementary data [file bmjqs-2023-016468supp002.pdf]

Appendix 2: MEDLINE search

Database: MEDLINE
Host: Ovid
Issue: 1946 to February 10, 2021
Date Searched: 12/2/2021
Searcher: SB
Hits: 405 (nurses); 40 (midwives); 6 (paramedics)
Strategy:

1. (nurse or nurses or nursing).tw.
2. nursing/
3. exp specialties, nursing/
4. or/1-3
5. (midwif* or midwives).tw.
6. Midwifery/
7. 5 or 6
8. paramedic*.tw.
9. Emergency Medical Technicians/
10. 8 or 9
11. ("mental health" or "mental ill health" or stress* or distress* or anxiety or anxious or depression or depressed or "wellbeing" or wellbeing or resilienc*).tw.
12. (pressure* adj3 (work* or "patient* demand*")).tw.
13. *Mental Health/
14. Stress, Psychological/
15. *Depression/
16. *Anxiety/
17. or/11-16
18. (retention or presenteeism or absenteeism or "sick leave" or burnout or "burn* out").tw.
19. Presenteeism/
20. *Absenteeism/
21. *Sick Leave/
22. or/18-21
23. exp United Kingdom/
24. (national health service* or nhs*).ti,ab,in.
25. (english not ((published or publication* or translat* or written or language* or speak* or literature or citation*) adj5 english)).ti,ab.
26. (gb or "g.b." or britain* or (british* not "british columbia") or uk or "u.k." or united kingdom* or (england* not "new england") or northern ireland* or northern irish* or scotland* or scottish* or ((wales or "south wales") not "new south wales") or welsh*).ti,ab,jw,in.
27. (bath or "bath's" or ((birmingham not alabama*) or ("birmingham's" not alabama*) or bradford or "bradford's" or brighton or "brighton's" or bristol or "bristol's" or carlisle* or "carlisle's" or (cambridge not (massachusetts* or boston* or harvard*)) or ("cambridge's" not (massachusetts* or boston* or harvard*)) or (canterbury not zealand*) or ("canterbury's" not zealand*) or chelmsford or "chelmsford's" or chester or "chester's" or chichester or "chichester's" or coventry or "coventry's" or derby or "derby's" or (durham not (carolina* or nc)) or ("durham's" not (carolina* or nc)) or ely or "ely's" or exeter or "exeter's" or gloucester or "gloucester's" or hereford or "hereford's" or hull or "hull's" or lancaster or "lancaster's" or leeds* or leicester or "leicester's" or (lincoln not nebraska*) or ("lincoln's" not nebraska*) or (liverpool not (new south wales* or nsw)) or ("liverpool's" not (new south wales* or nsw)) or ((london not (ontario* or ont or toronto*)) or ("london's" not (ontario* or ont or toronto*)) or manchester or "manchester's" or (newcastle not (new south wales* or nsw)) or

("newcastle's" not (new south wales* or nsw)) or norwich or "norwich's" or nottingham or "nottingham's" or oxford or "oxford's" or peterborough or "peterborough's" or plymouth or "plymouth's" or portsmouth or "portsmouth's" or preston or "preston's" or ripon or "ripon's" or salford or "salford's" or salisbury or "salisbury's" or sheffield or "sheffield's" or southampton or "southampton's" or st albans or stoke or "stoke's" or sunderland or "sunderland's" or truro or "truro's" or wakefield or "wakefield's" or wells or westminster or "westminster's" or winchester or "winchester's" or wolverhampton or "wolverhampton's" or (worcester not (massachusetts* or boston* or harvard*)) or ("worcester's" not (massachusetts* or boston* or harvard*)) or (york not ("new york*" or ny or ontario* or ont or toronto*)) or ("york's" not ("new york*" or ny or ontario* or ont or toronto*))))).ti,ab,in.

28. (bangor or "bangor's" or cardiff or "cardiff's" or newport or "newport's" or st asaph or "st asaph's" or st davids or swansea or "swansea's").ti,ab,in.
29. (aberdeen or "aberdeen's" or dundee or "dundee's" or edinburgh or "edinburgh's" or glasgow or "glasgow's" or inverness or (perth not australia*) or ("perth's" not australia*) or stirling or "stirling's").ti,ab,in.
30. (armagh or "armagh's" or belfast or "belfast's" or lisburn or "lisburn's" or londonderry or "londonderry's" or derry or "derry's" or newry or "newry's").ti,ab,in.
31. or/23-30
32. (exp africa/ or exp americas/ or exp antarctic regions/ or exp arctic regions/ or exp asia/ or exp australia/ or exp oceania/) not (exp United Kingdom/ or europe/)
33. 31 not 32
34. 4 and 17 and 22 and 33 [Nursing literature]
35. 7 and 17 and 22 and 33 [Midwifery literature]
36. 10 and 17 and 22 and 33 [Paramedics literature]
