# Supplementary material for: Care Under Pressure 2: a realist synthesis of causes and interventions to mitigate psychological ill health in nurses, midwives and paramedics
Source: BMJ Qual Saf. 2024 Apr 4;33(8):523–38. doi: 10.1136/bmjqs-2023-016468 (PMC11287552; doi:10.1136/bmjqs-2023-016468)
Supplement: Supplementary data [file bmjqs-2023-016468supp003.pdf]

Appendix 3: Included sources in the review

#	Author(s)	Title/citation	Year	Profession	Focus: Causes, Interventions, or Both	Type of paper/Approach (If empirical, brief method)
**Formal database searches**						
Nurses (n=23)						
1	Anderson, N.	An evaluation of a mindfulness-based stress reduction intervention for critical care nursing staff: A quality improvement project. *Nursing in Critical Care*, *26*(6), 441–448.	2021	Nurses	Interventions	Empirical / Quantitative (Intervention evaluation)
2	Andrews, H., Tierney, S. et al.	Needing permission: The experience of self-care and self-compassion in nursing: A constructivist grounded theory study. *International Journal of Nursing Studies*, *101*, 103436	2020	Nurses	Both	Empirical / Qualitative (Constructivist Grounded Theory)
3	Berry, S. & Robertson, N.	Burnout within forensic psychiatric nursing: Its relationship with ward environment and effective clinical supervision? *Journal of Psychiatric and Mental Health Nursing*, *26*(7–8), 212–222	2019	Nurses	Interventions	Empirical / Quantitative (Cross-sectional design)
4	Best, C.	Building resilience in contemporary nursing practice. *Practice Nursing*, *30*(8), 400–404.	2019	Nurses	Causes	Commentary
5	Brett Bowen, A.	How do emergency nurse practitioners experience managing acutely unwell patients in minor injury units? An Interpretative Phenomenological Analysis. *International Emergency Nursing*, *43*, 99–105	2019	Nurses	Causes	Empirical / Qualitative (Interpretative Phenomenological Analysis)
6	Cedar, S.H. & Walker, G.	Protecting the wellbeing of nurses providing end-of-life care. *Nursing Times*, 116(2), 30-34.	2020	Nurses	Interventions	Commentary
7	Chesterton, L., et al.	A hermeneutical study of professional accountability in nursing. *Journal of clinical nursing*, 30(1-2), 188-199.	2021	Nurses	Both	Empirical / Qualitative (Phenomenological design)
8	Delaney, M.C.	Caring for the caregivers: Evaluation of the effect of an eight-week pilot mindful self-compassion (MSC) training program on nurses' compassion fatigue and resilience. *PloS one*, 13(11), e0207261.	2018	Nurses	Interventions	Empirical / Mixed-methods (Observational study)
9	Duncan, M.	Managing sickness absence and declared disabilities in a district nursing team. *British Journal of Community Nursing*, *24*(10), 478–481.	2019	Nurses	Both	Commentary

#	Author(s)	Title/citation	Year	Profession	Focus: Causes, Interventions, or Both	Type of paper/Approach (If empirical, brief method)
10	Dunlop, S., & Maunder, E.Z.	Developing and nurturing a community practice clinical network for community children's nurses in Wales. *British Journal of Nursing*, *28*(12), 782–786.	2019	Nurses	Interventions	Commentary
11	Fasbender, U., Van der Heijden, B, & Grimshaw, S.	Job satisfaction, job stress and nurses' turnover intentions: The moderating roles of on-the-job and off-the-job embeddedness. *Journal of Advanced Nursing*, *75*(2), 327–337.	2019	Nurses	Causes	Empirical / Quantitative (Survey)
12	Goddard, D., et al.	Prison Nurses' Professional Identity. *Journal of Forensic Nursing*, *15*(3), 163–171.	2019	Nurses	Both	Discussion paper
13	Higgins, J.T., et al.	Factors Associated with Burnout in Trauma Nurses. *Journal of Trauma Nursing*, *27*(6), 319–326.	2020	Nurses	Causes	Empirical / Quantitative (secondary analysis)
14	Jackson, H.	Retaining and valuing newly qualified nursing staff: evaluation of a peer support group. Mental Health Practice, 21(8),24-27.	2018	Nurses	Interventions	Empirical / Qualitative (Service evaluation)
15	Laker, C., et al.	The impact of ward climate on staff perceptions of barriers to research-driven service changes on mental health wards: A cross-sectional study. *Journal of Psychiatric and Mental Health Nursing*, *27*(3), 281–295.	2020	Nurses	Causes	Empirical / Quantitative (Randomised Controlled Trial)
16	Laker, C., Cella, M., Callard, F., & Wykes, T.	Why is change a challenge in acute mental health wards? A cross-sectional investigation of the relationships between burnout, occupational status and nurses' perceptions of barriers to change. *International Journal of Mental Health Nursing*, *28*(1), 190–198.	2019	Nurses	Causes	Empirical / Quantitative (RCT)
17	Marran, E.J.	Supporting staff who are second victims after adverse healthcare events. *Nursing Management*, *26*(6), 36–43.	2019	Nurses	Both	CPD Exercise
18	O'Neill, L., Johnson, J. & Mandela, R.	Reflective practice groups: Are they useful for liaison psychiatry nurses working within the Emergency Department? *Archives of Psychiatric Nursing*, *33*(1), 85–92.	2019	Nurses	Interventions	Empirical / Qualitative (Thematic Analysis)
19	Rodriguez Santana, I., et al	The impact of extending nurse working hours on staff sickness absence: Evidence from a large mental health hospital in England. *International Journal of Nursing Studies*, *112*, 103611.	2020	Nurses	Both	Empirical / Quantitative (Causal Analysis)
20	Stacey, G., et al.	The implementation of resilience based clinical supervision to support transition to practice in newly qualified healthcare professionals. *Nurse Education Today*, *94*, 104564.	2019	Nurses	Interventions	Empirical / Qualitative (Service Evaluation)

#	Author(s)	Title/citation	Year	Profession	Focus: Causes, Interventions, or Both	Type of paper/Approach (If empirical, brief method)
21	Webster, N., et al.	Experiences of peer support for newly qualified nurses in a dedicated online group: Study protocol. *Journal of Advanced Nursing*, *75*(7), 1585–1591.	2019	Nurses	Interventions	Study Protocol / Qualitative
22	Whiting, L., et al.	Factors influencing nurse retention within children's palliative care. *Journal of Child Health Care*, *25*(4), 587–602.	2021	Nurses	Interventions	Empirical / Mixed-methods (Appreciative Inquiry Approach)
23	Younge, L. et al.	Regular clinical supervision to enhance wellbeing in inflammatory bowel disease specialist nurses: A small pilot study. *Gastrointestinal Nursing*, *18*(3), 36–42.	2020	Nurses	Interventions	Empirical / Qualitative (Exploratory Qualitative Approach)
Midwives (n=13)						
24	Byrne, I.,	Is the perfect midwife attainable in modern maternity care? MIDIRS *Midwifery Digest*, 28(1), 28-34.	2018	Midwives	Both	Commentary
25	Clarke, E.	Toasted, Fried or Frazzled? Burnout and stress in midwifery practice. *Midwifery Matters*, *Winter 2013*(139), 15–16	2013	Midwives	Interventions	Commentary
26	Copp, E. & Morton, N.	Attention: calm and relaxed midwives at work! *Practising Midwife*, 14(4), 21-23.	2011	Midwives	Interventions	Commentary
27	Cull, J., et al.	"Overwhelmed and out of my depth": Responses from early career midwives in the United Kingdom to the Work, Health and Emotional Lives of Midwives study. *Women and Birth*, *33*(6), e549–e557.	2020	Midwives	Causes	Empirical / Qualitative (Thematic Analysis)
28	Hunter, B., Fenwick, J., et al.	Midwives in the United Kingdom: Levels of burnout, depression, anxiety and stress and associated predictors. *Midwifery*, *79*, 102526	2019	Midwives	Causes	Empirical / Quantitative (Cross-sectional survey)
29	Iaschi, E.	*Evaluation of Preceptorship Programme for Newly Qualified Midwives*. MIDIRS Midwifery Digest, 30(2), 177-179.	2020	Midwives	Interventions	Grey Literature / Mixed-methods
30	Power, A.	Midwifery in the 21st century: Are students prepared for the challenge? *British Journal of Midwifery*, *24*(1), 66–68.	2016	Midwives	Both	Commentary
31	Rocca-Ihenacho, L., Yuill, C. & McCourt, C.	Relationships and trust: Two key pillars of a well-functioning freestanding midwifery unit. *Birth*, *48*(1), 104-113.	2021	Midwives	Interventions	Empirical / Qualitative (Critical Realist Ethnography)
32	Sheen, K., Spiby, H. & Slade, P.	Exposure to traumatic perinatal experiences and posttraumatic stress symptoms in midwives: prevalence and association with burnout. *International journal of nursing studies*, 52(2), 578-87.	2015	Midwives	Causes	Empirical / Quantitative (Survey)

#	Author(s)	Title/citation	Year	Profession	Focus: Causes, Interventions, or Both	Type of paper/Approach (If empirical, brief method)
33	Slade, P., et al.	A programme for the prevention of post-traumatic stress disorder in midwifery (POPPY): indications of effectiveness from a feasibility study. *European journal of psychotraumatology*, 9(1), 1518069.	2015	Midwives	Intervention	Empirical / Quantitative (Feasibility Study)
34	Warriner, S., Hunter, L., & Dymond, M.	Mindfulness in maternity: Evaluation of a course for midwives. *British Journal of Midwifery*, *24*(3), 188–195.	2016	Midwives	Interventions	Empirical / Quantitative (Survey)
35	Warwick, C.	Midwives can no longer keep services afloat: A recent survey has revealed that midwives are leaving the profession to escape excessive workloads. *Nursing Standard*, *31*(12), 27–27.	2016	Midwives	Both	Commentary
36	Yoshida, Y., & Sandall, J.	Occupational burnout and work factors in community and hospital midwives: A survey analysis. *Midwifery*, *29*(8), 921–926.	2013	Midwives	Interventions	Empirical / Quantitative (Survey)
Paramedics (n=3)						
37	McDonald, M.A., Meckes, S.J., & Lancaster, C.L.	Compassion for Oneself and Others Protects the Mental Health of First Responders. *Mindfulness*, *12*(3), 659–671.	2021	Paramedics	Both	Empirical / Quantitative (Online Survey)
38	Treglown, L., et al.	The Dark Side of Resilience and Burnout: A Moderation-Mediation Model. *PLOS ONE*, *11*(6), e0156279.	2016	Paramedics	Causes	Empirical / Quantitative (Online Survey)
39	Wild, J., et al.	A prospective study of pre-trauma risk factors for post-traumatic stress disorder and depression. *Psychological Medicine*, *46*(12), 2571–2582.	2016	Paramedics	Causes	Empirical / Quantitative (Online Survey)
**Supplementary hand search (n=30)**						
Midwifery (n=13)						
40	Anonymous Blog	A comment on kindness. British Journal of Midwifery, 2018. 26(11): p. 758-758.	2018	Midwives	Interventions	Commentary
41	Axcell, C.	Mental health and the midwife. *British Journal of Midwifery, 27*(6), 398-398.	2019	Midwives	Both	Commentary
42	Barker, K.	Giving midwives some ‘me’ time. *British Journal of Midwifery, 27*(4), 210- 210.	2019	Midwives	Both	Commentary
43	Barker, K.	Building bricks and resilience. *British Journal of Midwifery, 26*(12), 767-767.	2018	Midwives	Interventions	Commentary

#	Author(s)	Title/citation	Year	Profession	Focus: Causes, Interventions, or Both	Type of paper/Approach (If empirical, brief method)
44	Barker, K.	Reasons why midwives leave. *British Journal of Midwifery*, *24*(12), 826–826.	2016	Midwives	Both	Commentary
45	Brintworth, K.	Listening in: A survey of supervisors of midwives in London. *British Journal of Midwifery*, *22*(6), 432–437.	2014	Midwives	Interventions	Empirical / Mixed-methods (Online survey)
46	Golden, P.	Who has a duty of care to keep midwives safe? *British Journal of Midwifery*, *26*(1), 62–63	2018	Midwives	Interventions	Commentary
47	Leversidge, A.	Caring for midwifery staff will ensure better care for women. *British Journal of Midwifery*, *24*(7), 463–463.	2016	Midwives	Intervention	Commentary
48	Martin, C.J.H., et al.	Teaching compassionate mind training to help midwives cope with traumatic clinical incidents. *British Journal of Midwifery*, *29*(1), 26-35.	2020	Midwives	Interventions	CPD Exercise
49	Newman, L.	Bullying: the issue in (and beyond) midwifery. British Journal of Midwifery, 2019. 27(9): p. 541-541.	2019	Midwives	Causes	Editorial
50	Pezaro, S., Pearce, G., & Bailey, E.	Childbearing women's experiences of midwives' workplace distress: Patient and public involvement. *British Journal of Midwifery*, *26*(10), 659–669	2018	Midwives	Interventions	Empirical / Qualitative (PPI)
51	Wain, A.	Examining the lived experiences of newly qualified midwives during their preceptorship. *British Journal of Midwifery*, *25*(7), 451–457	2017	Midwives	Intervention	Empirical / Qualitative (Interpretive Phenomenological Analysis)
52	Winter, G.	Dealing with burnout. *British Journal of Midwifery*, *27*(12), 802-802.	2019	Midwives	Intervention	Commentary
Paramedics (n=17)						
53	Daubney, E.	Use of dark humour as a coping mechanism. *Journal of Paramedic Practice*, *11*(3), 128–128.	2019	Paramedics	Interventions	Commentary
54	Daubney, E.	Emotional resilience in the ambulance service. *Journal of Paramedic Practice*, *10*(12), 537-537.	2018	Paramedics	Interventions	Commentary
55	Gilroy, R.	Mental health: caring for the paramedic workforce. *Journal of Paramedic Practice*, *10*(5), 192-193.	2018	Paramedics	Interventions	Commentary
56	Johnston, S.	My time as a paramedic and why mental health matters. *Journal of Paramedic Practice*, *10*(7), 309-309.	2018	Paramedics	Interventions	Commentary
57	Mendes, A.	Are you 'enhanced' by your stress? *Journal of Paramedic Practice*, *12*(7), 261–261.	2020	Paramedics	Interventions	Editorial

#	Author(s)	Title/citation	Year	Profession	Focus: Causes, Interventions, or Both	Type of paper/Approach (If empirical, brief method)
58	Mendes, A.	How does your job impact your health? *Journal of Paramedic Practice*, *10*(9), 369-369.	2018	Paramedics	Interventions	Editorial
59	Mildenhall, J.	Protecting the mental health of UK paramedics. *Journal of Paramedic Practice*, *11*(1), 6-7.	2019	Paramedics	Both	Commentary
60	Miller, E.	The prevalence of stress and burnout in UK emergency ambulance service workers and its impact on their mental health and well-being. *Br Paramed J, 5*(4), 62-63.	2021	Paramedics	Causes	Conference Abstract / Mixed-methods (Online survey)
61	Miller, J.	'We wear too many caps': role conflict among ambulance service managers. *Br Paramed J, 3*(4), 44.	2019	Paramedics	Interventions	Conference Abstract / Qualitative (Semi-structured Interviews)
62	Naumann, D., et al.	Acute stress and frontline healthcare providers. *Journal of Paramedic Practice, 9*(12), 516-521.	2017	Paramedics	Causes	Empirical / Mixed-methods (Cross-sectional observation)
63	Paranjape, A.	Helping the helpers: how paramedics were cared for. *Journal of Paramedic Practice, 9*(6), 235-235.	2017	Paramedics	Both	Editorial
64	Paranjape, A.	Taking care of the practising paramedic. *Journal of Paramedic Practice, 8*(12), 575-575.	2016	Paramedics	Both	Editorial
65	Peate, I.	The workplace and mental wellbeing. *Journal of Paramedic Practice 9*(10), 419-419.	2017	Paramedics	Both	Commentary
66	Quaile, A.	Ambulance staff contemplate suicide due to stress and poor mental health. *Journal of Paramedic Practice, 8*(5), 224-226.	2016	Paramedics	Both	Commentary
67	Sibson, L.	Mental health in emergency care. *Journal of Paramedic Practice, 9*(9), 373-373.	2017	Paramedics	Both	Editorial
68	Smith, D.	What is your 'normal'? *Journal of Paramedic Practice*, *11*(2), 83.	2021	Paramedics	Interventions	Commentary
69	Van der Gaag, A., et al.	Why do paramedics have a high rate of self-referral? *Journal of Paramedic Practice*, *10*(5), 205-210.	2018	Paramedics	Interventions	Empirical / Mixed-methods
**Expert Solicitation (n=32)**						
70	Ball, J.E., et al.	'Care left undone' during nursing shifts: associations with workload and perceived quality of care. *BMJ quality & safety*, *23*(2), 116-125.	2014	Nurses	Causes	Empirical (quantitative) Questionnaire survey
71	Beryl, R., Davies, J & Völlm, B.	Lived experience of working with female patients in a high-secure mental health setting. *International Journal of Mental Health Nursing*, 27(1), 82-91. Moved from HS	2018	Nurses	Interventions	Empirical / Qualitative (Thematic Analysis)

#	Author(s)	Title/citation	Year	Profession	Focus: Causes, Interventions, or Both	Type of paper/Approach (If empirical, brief method)
72	Bosanquet, J.	Providing not prescribing: fostering a culture of wellbeing in nursing. Journal of Research in Nursing, 26(5): p. 367-375. Moved from HS	2021	Nurses	Both	Editorial
73	Churchill, L.R. & Schenck, D.	*Healing skills for medical practice. Annals of Internal Medicine, 149*(10), 720-4.	2008	Allopathic complementary and alternative medicine practitioners	To identify a core set of healing skills	Empirical (qualitative) Interviews
74	Davies, N.	*Vicarious trauma in nursing*, in *Independent Nurse*, (16 April 2021). Available from: https://www.independentnurse.co.uk/content/professional/vicarious-trauma-in-nursing/	2021	Nurses	Causes	Editorial
75	Epstein, E.G., et al.	Enhancing understanding of moral distress: the measure of moral distress for health care professionals. *AJOB empirical bioethics*, *10*(2), 113-124.	2019	All healthcare workers	Causes	Empirical Quantitative (Survey instrument development and testing)
76	Figley, C. R	Compassion fatigue: *Toward a new understanding of the costs of caring* in *Secondary traumatic stress: Self-care issues for clinicians, researchers, and educators*, B.H. Stamm, Editor. 1999, Sidran: Lutherville, MD. 3-28.	1995	All healthcare workers	Causes	Discursive review chapter
77	Gray, P., et al.	Workplace-Based Organizational Interventions Promoting Mental Health and Happiness among Healthcare Workers: A Realist Review. *Int J Environ Res Public Health, 16*(22), 4396.	2019	All healthcare workers	Both	Realist literature review
78	Gupta, A., Harris, D., & Naina, H.V.	The impact of physician posture during oncology patient encounters. *Journal Cancer Education, 30*(2), 395-7	2015	Doctors	Causes	Editorial/ reflection
79	Hewett, M.	Ethics and toxic high-workload work environments. *British Journal of General Practice, 72*(718), 226-227.	2022	Doctors	Causes	Editorial
80	Hillen, M.A., et al.	How can communication by oncologists enhance patients' trust? An experimental study. *Annals of Oncology, 25*(4), 896-901.	2014	Doctors	Causes	Empirical Quantitative Videotaped consultations assessed by patients and rated
81	Hochschild, A.,	*The Managed Heart: Commercialization of Human Feeling* 3rd ed. California: The University of California Press.	2012	All healthcare workers	Causes	Empirical (qualitative) Social constructivist interviews

#	Author(s)	Title/citation	Year	Profession	Focus: Causes, Interventions, or Both	Type of paper/Approach (If empirical, brief method)
82	Johns, G.	Presenteeism in the workplace: A review and research agenda. *Journal of organizational behavior*, *31*(4), 519-542.	2010	All healthcare workers	Causes	Narrative review
83	Jones, A., et al.	Interventions promoting employee "speaking-up" within healthcare workplaces: A systematic narrative review of the international literature. *Health Policy*, *125*(3), 375-384.	2021	All healthcare workers	Interventions	Systematic Narrative Review
84	Jones, A., & Kelly, D.	Deafening silence? Time to reconsider whether organisations are silent or deaf when things go wrong. *BMJ quality & safety*, *23*(9), 709-713.	2014	All healthcare workers	Causes	Editorial
85	Khatri, N., G.D. Brown, G.D & Hicks, L.L.	From a blame culture to a just culture in health care. *Health Care Management Review, 34*(4), 312-22.	2009	All healthcare workers	Causes	Conceptual review
86	Limb, M.	Need for accountability should not result in "toxic" blame culture in NHS, conference hears. *BMJ, 348*, g2282.	2014	All healthcare workers	Causes	Conference news report
87	Maben, J.	The art of caring: invisible and subordinated? A response to Juliet Corbin: 'is caring a lost art in nursing?'. *International journal of nursing studies*, *45*(3), 335-338.	2008	Nurses	Causes	Editorial
88	Maben, J., et al.	Living life in limbo: experiences of healthcare professionals during the HCPC fitness to practice investigation process in the UK. *BMC Health Services Research, 21*(1),839. Moved from HS	2021	Paramedics	Both	Empirical / Qualitative (Semi-structured Interviews)
89	Maben, J., et al.,	*A realist informed mixed-methods evaluation of Schwartz Center Rounds® in England.* 2018, NIHR: Southampton.	2018	All healthcare workers	Both	Empirical (mixed methods) Realist evaluation
90	Maben, J., Ball, J., & Edmondson, A.	*Workplace Conditions (Elements of Improving Quality and Safety in Healthcare)*. Cambridge: Cambridge University Press.	2022	All healthcare workers	Both	Narrative Review (book chapter)
91	Maben, J., Latter, S., & Clark, J.M.	The sustainability of ideals, values and the nursing mandate: evidence from a longitudinal qualitative study. *Nursing inquiry*, *14*(2), 99-113.	2007	Nurses	Causes	Empirical (qualitative) Semi-structured interviews
92	Maben, J., Latter, S., & Clark, J.M.	The theory–practice gap: impact of professional–bureaucratic work conflict on newly-qualified nurses. *Journal of Advanced Nursing*, (55), 465-477.	2006	Nurses	Causes	Empirical (qualitative) Semi-structured interviews
93	Maben, J., C. Taylor, et al.	Realist evaluation of Schwartz rounds® for enhancing the delivery of compassionate healthcare: understanding how they	2021	All healthcare workers	Both	Empirical / Qualitative (Realist Evaluation)

#	Author(s)	Title/citation	Year	Profession	Focus: Causes, Interventions, or Both	Type of paper/Approach (If empirical, brief method)
		work, for whom, and in what contexts. *BMC health services research*, *21*, 1-24.				
94	Menzies, I.E.P.	A case-study in the functioning of social systems as a defence against anxiety: a report on a study of the nursing service of a general hospital. *Human Relations, 13*(2), 95-121.	1960	Nurses	Causes	Empirical (qualitative)<br>Interviews and observations
95	Morley, G., Bradbury-Jones, C., & Ives, J.	What is 'moral distress' in nursing? A feminist empirical bioethics study. *Nursing Ethics, 27*(5), 1297-1314.	2019	Nurses	Causes	Empirical (Qualitative)<br>(phenomenological interviews)
96	Rowe, A. & Regehr, C.	Whatever Gets You Through Today: An Examination of Cynical Humor Among Emergency Service Professionals. *Journal of Loss and Trauma*, 15(5), 448-464. Moved from HS	2010	Paramedics	Interventions	Discussion Paper
97	Safazadeh, S., et al.	Exploring the reasons for theory-practice gap in emergency nursing education: A qualitative research. *Journal Education and Health Promotion, 7*, 132.	2018	Nurses	Causes	Empirical (qualitative)<br>Semi-structured interviews
98	Scott, J., et al.	Understanding Healing Relationships in Primary Care. *The Annals of Family Medicine, 6*(4), 315.	2008	Doctors	To identify a core set of healing skills	Empirical (qualitative)<br>Interviews
99	Sanford, N., et al	Capturing challenges and trade-offs in healthcare work using the pressures diagram: An ethnographic study. *Applied Ergonomics*, *101*, 103688.	2021	Nurses	Causes	Empirical / Qualitative<br>(Ethnography)
100	Sirriyeh, R., et al.	Coping with medical error: a systematic review of papers to assess the effects of involvement in medical errors on healthcare professionals' psychological well-being. *Quality and safety in health care*, *19*(6), e43-e43.	2010	All healthcare workers	Causes	Systematic review
101	Ulys, C., Carrieri, D. & Mattick, K.	The impact of shared social spaces on the wellness and learning of junior doctors: A scoping review. *Medical education*, *57*(4), 315–330.	2023	Doctors	Both	Scoping review
**Expert Solicitation – Reports (n=7)**						
102	David, D.	The 'Ambulance Burnout' Issue. The Larrey Society.	2015	Paramedics	Both	Report on findings of members survey
103	Hunter, B., Henley, J. & Fenwick, J.	Work, Health and Emotional Lives of Midwives in the United Kingdom: The UK WHELM study. School of healthcare Sciences, Cardiff University.	2018	Midwives	Both	Project report to Funder

Taylor C, *et al. BMJ Qual Saf* 2024; 33:523–538. doi: 10.1136/bmjqs-2023-016468

#	Author(s)	Title/citation	Year	Profession	Focus: Causes, Interventions, or Both	Type of paper/Approach (If empirical, brief method)	
104	Hunter, B & Warren, L.	Investigating resilience in midwifery. Cardiff University: Cardiff	2013	Midwives	Both	Project report to Funder	
105	Kinman, G., Teoh, K. & Harriss, A.	The Mental Health and Wellbeing of Nurses and Midwives in the United Kingdom. Society of Occupational Medicine.	2020	Nurses, Midwives	Both	Commissioned Report	
106	NHS Health Education England	NHS Staff and Learners' Mental Health Commission.	2019	Nurses, Midwives, Paramedics	Both	Commissioned Report	
107	Royal College of Nursing	10 Unsustainable Pressures on the Health and Care System in England.	2021	Nurses, Midwives, Paramedics	Both	Report by Royal College of Nursing	
108	West, M., Bailey, S. & Williams, E.	The courage of compassion: supporting nurses and midwives to deliver high-quality care. The King's Fund: London.	2020	Nurses, Midwives	Both	Commissioned Report	
109	Carrieri, D., Pearson, M., Mattick, K.,et al	Interventions to minimise doctors’ mental ill-health and its impacts on the workforce and patient care: the care under pressure realist review. *Health Serv Deliv Res*	2020	Doctors	Both	Project report to Funder.	
**Literature Reviews (n=29)**							
Database Search (n=19)							*Number of included papers (UK)*
110	Albendin-Garcia, L., et al.	Prevalence, Related Factors, and Levels of Burnout Among Midwives: A Systematic Review. *Journal of midwifery & women's health*.	2021	Midwives	Causes	Systematic Review	27 (4)
111	Aryankhesal, A., et al.	Interventions on reducing burnout in physicians and nurses: A systematic review. *Medical journal of the Islamic Republic of Iran*, 33:77.	2019	Nurses	Interventions	Systematic Review	18(2)
112	Bacchus, A. & Firth, A.	What factors affect the emotional well-being of newly qualified midwives in their first year of practice? *MIDIRS Midwifery Digest, 27*(4), 444-450.	2017	Midwives	Both	Systematic Review	4(2)
113	Bresesti, I, Folgori, L., & De Bartolo, P.	Interventions to reduce occupational stress and burn out within neonatal intensive care units: a systematic review. *Occupational and environmental medicine, 77*(8), 515-519.	2020	Nurses	Both	Systematic Review	6(1)

#	Author(s)	Title/citation	Year	Profession	Focus: Causes, Interventions, or Both	Type of paper/Approach (If empirical, brief method)	
114	Buckley, L., et al.	What is known about paediatric nurse burnout: a scoping review. *Human Resources for Health, 18*(1), 1-23.	2020	Nurses	Both	Scoping Review	78(0)
115	Chamanga, E., et al.	Factors influencing the recruitment and retention of registered nurses in adult community nursing services: an integrative literature review. *Primary health care research & development, 21*, e31.	2020	Nurses	Causes	Integrative Review	10(1)
116	Cummings, G., et al.	Leadership styles and outcome patterns for the nursing workforce and work environment: A systematic review. *International Journal of Nursing Studies*, *85*, 19-60.	2018	Nurses	Both	Systematic Review	129(0)
117	Elliott-Mainwaring, H.	How do power and hierarchy influence staff safety in maternity services? *British Journal of Midwifery, 29*(8), 430-439.	2021	Midwives	Both	Systematic Narrative Review	10(np)
118	Freeling, M., Rainbow, J., & Chamberlain, D.	Painting a picture of nurse presenteeism: A multicountry integrative review. *International Journal of Nursing Studies*, 109.	2020	Nurses	Causes	Integrative Review	17(0)
119	Gribben, L. & Semple, C.	Factors contributing to burnout and work-life balance in adult oncology nursing: An integrative review. *European journal of oncology nursing: the official journal of European Oncology Nursing Society, 50,* 101887.	2020	Nurses	Both	Integrative Review	20(0)
120	Hawkins, N., Jeong, S., & Smith, T.	New graduate registered nurses' exposure to negative workplace behaviour in the acute care setting: An integrative review. International Journal of Nursing Studies, 93,41-54.	2019	Nurses	Both	Integrative Review	16(0)
121	Hunter, L.	Making time and space: the impact of mindfulness training on nursing and midwifery practice. A critical interpretative synthesis. *Journal of clinical nursing, 25*(7-8), 918-29.	2016	Midwives	Both	Critical Interpretive Synthesis	5(0)
122	McDermid, F., Judy, M., & Peters, K.	Factors contributing to high turnover rates of emergency nurses: A review of the literature. *Australian critical care: official journal of the Confederation of Australian Critical Care Nurses, 33(4),* 390-396.	2020	Nurses	Causes	Narrative Review Thematic Analysis	20(0)
123	Oates, J., et al.	An integrative review of nursing staff experiences in high secure forensic mental health settings: Implications for recruitment and retention strategies. *Journal of advanced nursing, 76*(11), 2897-2908.	2020	Nurses	Causes	Integrative Review	15(6)

Taylor C, *et al. BMJ Qual Saf* 2024; 33:523–538. doi: 10.1136/bmjqs-2023-016468

#	Author(s)	Title/citation	Year	Profession	Focus: Causes, Interventions, or Both	Type of paper/Approach (If empirical, brief method)	
124	Rajamohan, S., Porock, D., & Chang, Y.	Understanding the Relationship Between Staff and Job Satisfaction, Stress, Turnover, and Staff Outcomes in the Person-Centered Care Nursing Home Arena. *Journal of nursing scholarship: an official publication of Sigma Theta Tau International Honor Society of Nursing, 51*(5), 560-568.	2019	Nurses	Both	Integrative Review	11(0)
125	Stacey, G., & Cook, G.	A scoping review exploring how the conceptualisation of resilience in nursing influences interventions aimed at increasing resilience. *International Practice Development Journal, 9*(1), 1-16.	2019	Nurses	Interventions	Scoping Review	16(2)
126	Webster, N., et al.	Using technology to support the emotional and social well-being of nurses: A scoping review protocol. *Journal of advanced nursing, 76*(1), 109-120.	2019	Nurses	Interventions	Scoping Review	11(2)
127	Welford, C.	What factors influence professional burnout in mid wives? MIDIRS *Midwifery Digest*, *28*(1), 35-40.	2018	Midwives	Both	Narrative Review	4(0)
128	Yu, F., et al.	Personal and work-related factors associated with nurse resilience: A systematic review. *International Journal of Nursing Studies*, 93, 129-140.	2019	Nurses	Causes	Systematic Review	28(np)
Hand Search (n=4)							
129	Brooks, D. & Brooks, R.	A systematic review: what factors predict Post-Traumatic Stress Symptoms in ambulance personnel? *British Paramedic Journal, 5*(4),18-24.	2021	Paramedics	Causes	Systematic Review	18(np)
130	Clark, L., et al.	Mental health, well-being and support interventions for UK ambulance services staff: an evidence map, 2000 to 2020. *British Paramedic Journal, 5*(4), 25-39.	2021	Paramedics	Both	Evidence Mapping Methodology	45(45)
131	Dodd, G.	PTSD, available support and development of services in the UK Ambulance Service. *Journal of Paramedic Practice, 9*(6), 258-263.	2017	Paramedics	Both	Methodical literature search strategy	np
132	Lancaster, C. & Philips, P.	How does the use of humour in the UK ambulance service affect a clinician's well-being? *British Paramedic journal, 6*(2), 26-33.	2021	Paramedics	Both	Thematic Analysis	4(np)
Expert solicitation (n=6)							

#	Author(s)	Title/citation	Year	Profession	Focus: Causes, Interventions, or Both	Type of paper/Approach (If empirical, brief method)	
133	Anderson, L.	The impact of paramedic shift work on the family system: a literature review. *College of Paramedics, 3*(4), 43.	2019	Paramedics	Both	Systematic Search Strategy	22(np)
134	Auth, N.M., et al.	Mental health and help seeking among trauma-exposed emergency service staff: a qualitative evidence synthesis. *BMJ Open, 12*(2), e047814.	2022	Paramedics	Both	Qualitative Evidence Synthesis	24 (5)
135	Barleycorn, D.	Awareness of secondary traumatic stress in emergency nursing. *Emergency nurse: the journal of the RCN Accident and Emergency Nursing Association, 27*(5),19-22.	2019	Nurses	Both	Narrative Review	12(np)
136	Brand, S., et al.	Whole-system approaches to improving the health and wellbeing of healthcare workers: A systematic review. *PLoS One, 12*(12), e0188418.	2017	Whole NHS	Interventions	Systematic Review	11(1)
137	Ejebu, O.Z., Dall'Ora, C., & Griffiths, P.	Nurses' experiences and preferences around shift patterns: A scoping review. *PLoS One, 16*(8), e0256300.	2021	Nurses	Causes	Scoping Review	30 (7)
138	Foster, A., Wood, E., & Clowes, M.	Identifying the evidence base of interventions supporting mental health nurses to cope with stressful working environments: A scoping review. *Journal of Nursing Management, 29(6),* 1639-1652.	2021	Nurses	Both	Scoping Review	18(3)
**COVID-19 Specific literature**							
Transferred from original search (n=2)							
139	Mendes, A.	Protecting your mind amid crisis. Journal of Paramedic Practice, 2020. 12(5): p. 177- 177.	2021	Paramedics	Interventions	Commentary	
140	Riedel, P.L., et al.	A scoping review of moral stressors, moral distress and moral injury in healthcare workers during COVID-19. *International Journal of Environmental Research and Public Health*, *19*(3), 1666.	2022	All healthcare workers	Causes	Scoping Review	
Database (n=12)							
141	Brooks, D.	Acknowledge Pandemic-Driven Moral Distress, Mitigate Harmful Effects. *ED Management, 33*(11), 1-2.	2021	Nurses	Causes	Discussion Paper	
142	Choflet, A., et al.	The Nurse Leader's Role in Nurse Substance Use, Mental Health, and Suicide in a Peripandemic World. *Nursing Administration Quarterly, 46*(1),19-28.	2022	Nurses	Both	Discussion paper	

#	Author(s)	Title/citation	Year	Profession	Focus: Causes, Interventions, or Both	Type of paper/Approach (If empirical, brief method)
143	Gardiner, M., DeMuy, A., & Tran, N.K.	Here4HealthCare: A Response to the Emerging Mental Health Crisis of the Frontline Healthcare Workforce. *Canadian Journal of Community Mental Health, 39*(3): 85-88.	2020	All healthcare workers	Interventions	Commentary
144	Hayes, C., Corrie, I & Graham, Y.	Paramedic emotional labour during COVID-19. *Journal of paramedic practice*, *12*(8), 319-323.	2021	Paramedics	Causes	Commentary
145	Llop-Gironés, A., et al.	Employment and working conditions of nurses: where and how health inequalities have increased during the COVID-19 pandemic? *Human Resources Health, 19*(112), 1-11.	2021	Midwives; Nurses	Causes	Literature Review
146	Maloney, L., et al.	MINDING THE MIND OF EMS, PART 2: Here's how to help employees' mental well-being. *EMS World, 50*(9), 36-41.	2021	Paramedics	Causes	Discussion Paper
147	Maloney, L., Hoffman, J., & Pepe, P.	Maloney, L., J. Hoffman, and P. Pepe, MINDING THE MIND OF EMS, PART I EMS World, November 2020.	2020	Paramedics	Causes	Discussion Paper
148	McFadden, P., et al.	The Role of Coping in the Wellbeing and Work-Related Quality of Life of UK Health and Social Care Workers during COVID-19. *International journal of environmental research and public health, 18*(2).	2021	All healthcare workers	Interventions	Empirical / Quantitative (Survey)
149	Rees, N., et al.	Paramedic experiences of providing care in Wales (UK) during the 2020 COVID-19 pandemic (PECC-19): a qualitative study using evolved grounded theory. *BMJ open, 11*(6), e048677.	2021	Paramedics	Causes	Empirical / Qualitative (Interviews)
150	Renger, A., Day, E., & Whitfield, S.	Describing a 12-hour ambulance shift during a second wave of COVID-19 in London. *Australasian Journal of Paramedicine, 18*, 1-4.	2021	Paramedics	Causes	Commentary
151	Teoh, K., Kinman, G., & Harriss, A.	Supporting nurses and their mental health in a world after Covid-19. *Occupational Health & Wellbeing, 72*(8),26-29.	2020	Nurses; Midwives	Causes	Editorial
152	Uytenbogaardt, A.	COVID-19's effect on midwives' mental health. *British Journal of Midwifery, 28*(6), 337-337.	2020	Midwives	Causes	Editorial
Expert solicitation (n=15)						
153	Abrams, R., et al.	Speaking up during the COVID-19 pandemic: Nurses' experiences of organizational disregard and silence. *Journal of Advanced Nursing*, 79, 2189– 2199.	2023	Nurses	Causes	Empirical (qualitative) Semi-structured interviews

#	Author(s)	Title/citation	Year	Profession	Focus: Causes, Interventions, or Both	Type of paper/Approach (If empirical, brief method)
154	Adams, M., et al.	Adams, M., et al., *How do "heroes" speak up? NHS staff raising concerns during Covid-19*. BMJ Opinion August 11th 2020. https://blogs.bmj.com/bmj/2020/08/11/how-do-heroes-speak-up-nhs-staff-raising-concerns-during-covid-19/	2020	All healthcare workers	Causes	Editorial
155	Blake, H., et al.	COVID-Well Study: Qualitative Evaluation of Supported Wellbeing Centres and Psychological First Aid for Healthcare Workers during the COVID-19 Pandemic. *International Journal Environment Research Public Health, 18*(7).	2021	All healthcare workers	Interventions	Empirical / Qualitative (Interviews)
156	Brooks, D.	Acknowledge Pandemic Driven Moral Distress, Mitigate Harmful Effects. *ED Management, 33*(11), 1-2.	2021	Nurses	Causes	Commentary
157	Conolly, A. Maben, J., et al.	'There was a pivotal moment'. The dynamics, transitions, adaptions and trajectories of nursing at the front-line in the UK during the COVID-19 pandemic: A longitudinal qualitative study. PLOSONE. 2023.	In press	Nurses	Causes	Empirical (qualitative) Semi-structured interviews
158	Dowrick A, Mitchinson L., et al.	Re-ordering connections: UK healthcare workers' experiences of emotion management during the COVID-19 pandemic. *Social Health Illn*;**43**(9):2156–77.	2021	All healthcare workers	Causes	Empirical (qualitative) Interviews
159	Greenberg, N., et al.	Mental health of staff working in intensive care during Covid-19. *Occupational Medicine, 71*(2),62-67.	2021	All healthcare workers	Causes	Empirical / Quantitative (Survey)
160	Greenberg, N.	"Going for Growth" An outline NHS staff recovery plan post-COVID19 (outbreak 1), Royal College of Psychiatrists. Available from: https://www.rcpsych.ac.uk/docs/default-source/about-us/covid-19/going-for-growth-version-3-05-05-20.pdf?sfvrsn=7cf71c97_4	2020	All healthcare workers	Interventions	Editorial / Guidance
161	Maben, J. & Conolly, A.	Lessons for structure, workplace planning and responding to emergencies from nurses in the COVID-19 pandemic. In R. Williams, V. Kemp, K. Porter, T. Healing & J. Drury (Eds.), *Pandemics, Major Incidents and Mental Health: The Psychosocial and Mental Health Aspects of Health Emergencies*. Cambridge: Cambridge University Press. In press.	In press	Nurses	Both	Empirical / Qualitative (Interviews)
162	Maben, J., et al.	"You can't walk through water without getting wet" Exploring nurse distress and psychological health needs during COVID-19:	2022	Nurses	Both	Empirical / Qualitative (Interviews)

#	Author(s)	Title/citation	Year	Profession	Focus: Causes, Interventions, or Both	Type of paper/Approach (If empirical, brief method)
		A longitudinal qualitative study, *International Journal of Nursing Studies, 131*, 104242.				
163	Maben, J., & Bridges, J.	Covid-19: Supporting nurses' psychological and mental health. *Journal of clinical nursing*, *29*(15-16), 2742.	2020	Nurses	Causes	Editorial
164	San Juan, N.V., et al.	Training and redeployment of healthcare workers to intensive care units (ICUs) during the COVID-19 pandemic: a systematic review. *BMJ open*, *12*(1), e050038.	2022	Doctors	Both	Empirical / Qualitative (Interviews)
165	Ustun, G.	COVID-19 Pandemic and Mental Health of Nurses: Impact on International Health Security. Contemporary Developments and Perspectives in International Health Security, ed. S. P. Stawicki et al. (eds.). Vol. Volume 2., London: IntechOpen.	2021	Nurses	Both	Narrative Review
166	Williams et al.	The top ten messages for supporting health care staff during the COVID-19 pandemic. Available from: https://aneurinbevanwellbeing.co.uk/wp-content/uploads/2020/10/Top-10-tips-to-support-health-care-staff-during-COVID19.pdf	2020	All healthcare workers	Both	Discussion Paper
167	Williamson, V., Murphy, D., & Greenberg, N.	COVID-19 and experiences of moral injury in frontline key workers. *Occupational Medicine, 70*(5), 317-319.	2020	All healthcare workers	Causes	Editorial

Taylor C, *et al. BMJ Qual Saf* 2024; 33:523–538. doi: 10.1136/bmjqs-2023-016468
