# Supplementary material for: Care Under Pressure 2: a realist synthesis of causes and interventions to mitigate psychological ill health in nurses, midwives and paramedics
Source: BMJ Qual Saf. 2024 Apr 4;33(8):523–38. doi: 10.1136/bmjqs-2023-016468 (PMC11287552; doi:10.1136/bmjqs-2023-016468)
Supplement: Supplementary data [file bmjqs-2023-016468supp004.pdf]

Appendix 4: Key findings, Tensions and CMOcs

Key finding	Tension	CMO number	Context-Mechanism-Outcome Configuration
It is difficult to promote staff psychological wellness where there is a blame culture	A lack of collective accountability vs a team/system-based approach	1	As frontline staff are most directly linkable to outcomes and errors (C), a focus on performance measurement and accountability of individual staff can preclude acceptance of system-wide accountability or attribution of errors (M-Resource), leading to staff practicing defensively to avoid blame (M-response), reducing job satisfaction, autonomy, quality of patient care and increasing risk of secondary trauma (O)
		2	When a clinical area has poor standards of care due to systemic/resourcing issues (C), if managers lack accountability, attention or ability to fix problems (M- Negative Resource), then staff feel angry at the double standards as they are professionally accountable for their actions (M-response), leading to job dissatisfaction, frustration, stress, burnout and intention to leave/attrition (O).
		3	When investigation of medical errors focusses on the individual and does not take account of wider context (C), then protracted regulatory and organisational policies that may suspend staff from work (M- Negative Resource), may cause staff to feel guilty, unsupported and isolated (M-Response), leading to risk of secondary trauma, suicide ideation and trauma extending to family/friends (O)
	Needing to raise concerns to improve conditions and patient safety vs fitness to practice processes becoming an oppressive force	4	When fitness-to-practice processes are not perceived or experienced as being supportive (C), then they may not be accessed when staff experience poor psychological health (M- Negative Resource), for fear of losing their status, reputation or employment (M-Response), so issues may remain undisclosed and unchecked, leading to worsening psychological health in staff, and increased risk of patient safety events (O)
		5	When medical errors occur in an organisation where staff do not feel psychologically safe (C) then investigation of errors (M-Resource) may make staff feel unheard or blamed, and they may fear public exposure and reputational damage, and not able to speak up and instead feel guilt and shame (M-Response), leading to silencing, frustration and secondary trauma (O)

Key finding	Tension	CMO number	Context-Mechanism-Outcome Configuration
	Encouraging staff to speak up vs the 'deaf effect' response from managers and hearers	6	In an organisation where it is not psychological safe to speak up about mistakes and errors or where senior leaders to do listen to staff concerns (C) when encouraged to speak up and raise concerns to identify and prevent harm (M-Resource), staff will fear the consequences or feel there is no point as no change will result (M-response), leading to decreased workplace satisfaction, poor staff retention, reduced quality of patient care, increased secondary trauma, stress and helplessness (O)
		7	In a healthcare system where approaches to support staff to deal with challenges at work are fragmented and non-systemic, leading managers not knowing where to take concerns for actions (C), then interventions that give permission for staff to voice concerns (M-Resource) can lead to managers feeling burden, frustration and guilt and to staff feeling isolated, abandoned and frustrated (M-response), causing reduced job satisfaction and engagement in staff and managers; reduced likelihood of speaking up in staff and increased risk of psychological ill-health and lower quality patient care (O).
'Serve and sacrifice': the needs of the system often override staff wellbeing at work	A professional culture in which staff prioritise institutional needs, vs a culture that promotes self-care	8	When high workloads without breaks become normalised in professions that are exhorted to put patients first (C) if messages from managers are that staff should give 100% to serve patients without providing support strategies for staff (M- Negative Resource), this reinforces compliance to institutional needs and a serve and sacrifice professional ethos to the detriment of staff needs (M-Response), leading to guilt, increased stress, burnout and intention to leave/attrition (O).
	Supporting existing staff in the context of staff shortages vs perceived coercion to fill 'extra' vacant shifts	9	In a healthcare service where managers feel pressure to ensure safe staffing levels despite staff shortages (C), when managers communicate this pressure to staff 'begging' them to work extra shifts (M- Negative Resource) staff can feel coerced and/or guilty when they say no (M-Response), preventing non-work time from being regenerative, causing work-related stress even when not at work, leading to increased job dissatisfaction, presenteeism and burnout (O).
	The lived reality of staff shortages vs the wish to deliver high quality care	10	When staff shortages mean there is less time to care for each patient (C), staff cannot provide their preferred quality of care (M- Negative Resource) leaving them feeling frustrated, dissatisfied, angry and guilty at the care left undone (M-Response), leading to moral distress and injury, burnout, intention to leave/attrition (O)
		11	In a stressful under-resourced work environment (C) more staff leave the professions/workplace (M- Negative Resource) creating more stress and pressure for those who remain (M-Response), leading to job dissatisfaction and burnout, and further attrition, creating a vicious cycle of staff depletion (O)

Key finding	Tension	CMO number	Context-Mechanism-Outcome Configuration
		12	When there are insufficient staff to perform all roles/tasks (C) remaining staff may not be as well supported or trained in their role or be asked to perform new task beyond the scope of their practice/grade (M- Negative Resource), causing anxiety and concern about quality of care and potential mistakes (M-Response), increasing stress, risk of burnout, sickness absence and intention to leave/attrition (O)
There are unintended personal costs of upholding and implementing values at work	The reality of healthcare delivery vs the taught theory and values	13	If newly qualified staff have developed idealised visions of work during training that espouses high ideals(C) then when pressures caused by staff shortages and other systemic factors mean their practice may not align with such ideals (M- Negative Resource), they may feel emotional and moral distress (M-Response), causing reduced job satisfaction, stress, burnout and intention to leave/attrition (O)
	The benefits of staff empathy to patient's vs the harms of staff empathy for themselves	14	If staff are recruited based on values including compassion, which requires staff to be empathic (C) when they are genuinely empathic (M-Resource) they are better able to understand the pain and suffering of patients (M-Response1) leading to better patient care, increased job satisfaction (O1) but empathising with patient suffering may also cause staff distress (M-Response2), leading to emotional exhaustion, burnout, secondary/vicarious trauma and staff leaving the professions (O2)
		15	If staff do not receive support for the emotional labour intrinsic to their work (C) then everyday pressures may deplete their empathy (M-Resource) causing staff to engage in maladaptive strategies to buffer against secondary trauma (M-Response), causing worsening of secondary traumatic stress symptoms, reduced quality of patient care, decreased job satisfaction and further burnout (O)
	The emotional labour inherent in healthcare practice vs the need to improve workplace psychological ill-health	16	Healthcare staff may be exposed to injuries or suffering that evokes natural emotions such as repulsion, fear or distress (C), but have to repress responses to protect patients (M-Resource) which can lead to emotional distress in staff (M-Response), causing suppressed emotions to come out in other dysfunctional ways, impacting job satisfaction, performance and psychological health (O)

Key finding	Tension	CMO number	Context-Mechanism-Outcome Configuration
Interventions are fragmented, individual-focussed and insufficiently recognise cumulative chronic stressors	A focus on individuals vs a focus on systemic issues.	17	In a workplace with staff and resource shortages (C) when staff are offered individual-focused wellbeing interventions in the absence of a systematic approach to wellbeing (M-Resource), they may feel that the stress they experience is their fault and responsibility to resolve (M-response), leading to staff feeling they have failed when they are unable to cope/do their jobs as expected, and/or feeling let down by their employer and to them disengaging with work or leaving the profession (O)
		18	When there is normalisation of unpaid overtime and in the absence of a systemic focus on wellbeing (C) if leaders/managers send messages encouraging staff to prioritise self-care (M-Resource) staff may feel that managers are out of touch with reality and ignore such messages (M-Response) leading to reduced job satisfaction, work engagement and morale (O)
		19	Staff are exhorted to put patients first and hide their own needs and emotions (C) but when permission to be self-compassionate is role-modelled or granted by managers and peers (M-Resource) staff can acknowledge importance of self-compassion (M-Response), leading to better work practices and caretaking of self, reduced stress, improved work satisfaction and compassion for other staff and patients (O).
	A focus on acute episodes of trauma vs recognising and supporting chronic cumulative stressors	20	Constant low-grade trauma exposure to patient suffering, resource scarcity and staff shortages may not be visible (C) meaning that managers may not recognise the cumulative build-up of stress (M-Negative Resource) and may thereby judge staff competency unfairly (M-Response) causing increased stress, risk of secondary trauma, and intention to leave/attrition in staff (O)
		21	If chronic low-grade trauma is not recognised (C) the need for support may be missed and chronic stress can become normalised (M- Negative Resource) leading to staff feeling insecure and inadequate, or failing to recognise signs of low-grade trauma in themselves or others (M-Response), which can result in staff finding ways to cope and carry on, worsening symptoms of stress/distress, higher risk of mistakes, sickness absence and intention to leave/attrition (O)

Key finding	Tension	CMO number	Context-Mechanism-Outcome Configuration
It is challenging to design, identify and implement interventions to work optimally for diverse staff groups	Making staff wellness interventions mandatory vs making them voluntary	22	In a healthcare organisation that wants to prioritise staff wellbeing (C) managers may mandate attendance at a wellness intervention when some staff are not receptive to it (M-Resource) leading to some staff feeling that the approach is tick-box and lacking authenticity, and may be left feeling resentful, anxious, exposed or stigmatised at sharing emotions (M-Response) causing staff to disengage from work, feel less secure and less likely to speak up (O).
		23	In a healthcare organisation that wants to prioritise staff wellbeing but recognises that staff need to engage in different ways with support (C) managers may offer debriefs or check-ins as a voluntary/optional intervention (M-Resource) meaning that staff that engage may feel supported (M-Response1), but others who need support may not engage due to not recognising the need or fearing stigma (M-Response2), causing variable provision of support where some staff benefit and feel supported and their concerns are heard but others may not, leading to increased stress, and psychological ill-health (O)
	The need for spaces to debrief with managers/leaders vs the need for peer-led spaces for debriefing	24	When staff are exposed to trauma or other stressors (C) managers may offer formal debriefing that aims to provide support and for organisational learning (M-Resource), but staff may feel that it is a management/performance tool, and not feel safe to disclose and feel unsupported (M-Response) leading to breakdown of trust, reduced disclosure and further exacerbation of stressors/trauma (O).
		25	Healthcare staff may face chronic and acute trauma exposure (C). When mentors offer kindness, listening and spaces to be heard (M-Resource), staff feel supported and that their experiences are important and recognised (M-Response), and are thereby helped to recover and heal, continue with work, feel less alone and protected from further harm (O)
	The need to act and offer support vs providing interventions that are ineffective because they are too soon, reactive and/or single timepoint	26	If staff basic physiological and safety needs are not met (C) then when they are offered other support/interventions such as end of shift debriefing (M-Resource) they may feel frustrated and upset due to the lack of recognition of their other essential needs, and fatigue and exhaustion due to intense working shifts preventing attendance (M-Response) causing low uptake /engagement and exacerbation of distress/trauma response (O)
